# Effects of Tropical Laterite Soils on Larval Development and the Metabolome of the Forensically Important Blow Fly *Chrysomya megacephala* (Diptera: Calliphoridae): An Exploratory Study

**DOI:** 10.3390/insects17080841

**Published:** 2026-08-13

**Authors:** Yuling Liu, Boqing Fan, Yixin Ma, Jianghuan Lu, Liwen Yin, Jiayao Xu, Siwei Ye, Yang Gao, Bo Wang, Jifeng Cai, Jianhua Chen, Jianqiang Deng

**Affiliations:** 1 Key Laboratory of Tropical Translational Medicine of Ministry of Education, School of Basic Medical Sciences, Hainan Academy of Medical Sciences, Hainan Medical University, Haikou 571199, China; lyluia1115@163.com (Y.L.); q202411122@163.com (B.F.); xmbebrave@163.com (Y.M.); lujh1127@163.com (J.L.); yhx20230202@126.com (L.Y.); xujy0806@163.com (J.X.); yes206206@163.com (S.Y.); gaoy2006@163.com (Y.G.); 2 Department of Forensic Medicine, Hainan Provincial Tropical Forensic Engineering Research Center, School of Basic Medical Sciences, Hainan Medical University, Haikou 571199, China; 3 Department of Forensic Medicine, School of Basic Medicine, Xinjiang Medical University, Ürümqi 830054, China; sapphirestrings@sina.com; 4 Department of Forensic Medicine, School of Basic Medicine, Central South University, Changsha 410083, China; cjf_jifeng@163.com

**Keywords:** forensic entomology, *Chrysomya megacephala*, laterite soil, postmortem interval, larval development, untargeted metabolomics, heavy metals, UHPLC-MS/MS

## Abstract

Laterite is an acidic tropical soil enriched in iron and manganese. It is common at tropical death scenes, yet its effect on necrophagous insect development is unstudied. We raised the forensically important blow fly *Chrysomya megacephala* (Fabricius, 1794) on three Hainan laterites, a yellow earth control, and a no-soil substrate. We combined larval phenotyping, inductively coupled plasma mass spectrometry (ICP-MS), and untargeted ultra-high-performance liquid chromatography–tandem mass spectrometry (UHPLC-MS/MS) metabolomics of whole larvae sampled on Days 1–3 post-burrowing. Laterites were strongly acidic (pH 4.17–4.32 vs. 7.45) and Fe/Mn-enriched. Whole-larval Fe (~10.2-fold) and Mn (~4.1-fold; whole larvae including gut contents, as no gut evacuation was performed) were consistently elevated across the three laterites, without biomagnification (bioaccumulation factors < 1). Laterite-group larvae were significantly heavier from 106 h and shorter from 130 h of age. They entered the cryptocephalic stage at 130 vs. 154 h and pupariated 24 h earlier in shallower soil (3–5 vs. 8–10 cm). In the primary merged metabolome analysis (*n* = 5–6 per group; 2975 metabolites), no metabolite survived the Benjamini–Hochberg correction in any laterite comparison, whereas three did in the no-soil comparison. The level-1 candidate metabolites are therefore hypothesis-generating. Laterite thus alters larval development and whole-body metal content. This implies a potential ~1-day bias in postmortem interval estimation under controlled conditions.

## Simple Summary

After death, investigators estimate the time since death by studying insects on the body. The tropical blow fly *Chrysomya megacephala* (Fabricius, 1794) is among the most important of these insects. After feeding, its maggots crawl into the surrounding soil to pupate. In hot, humid regions such as Hainan Island, China, the ground is often laterite, a red, acidic, iron-rich clay soil. Whether this soil changes fly growth had never been tested. In this study, we raised maggots on three laterite soils, on a paler reference soil (yellow earth), or with no soil. We measured their growth, body metal content, and internal small molecules during the first three days after burrowing. Laterite-reared maggots grew heavier from 106 h and shorter from 130 h. They began transforming into pupae about one day earlier and stayed in shallower soil. Their bodies, including gut contents (no gut evacuation was performed), contained ten times more iron and four times more manganese, without concentrating these metals above soil levels. Chemical profiling suggested changes in energy-metabolism molecules, but none survived strict statistical testing. These molecules remain clues for future work, not proven effects. Under controlled laboratory conditions, laterite could shift insect-based estimates of time since death by about one day. Field studies are needed first.

## 1. Introduction

Estimating the postmortem interval (PMI) is a central task of forensic investigation. Necrophagous insects, above all blow flies (Diptera: Calliphoridae), provide the most widely used biological clock [1,2]. Their development proceeds at temperature-dependent, largely predictable rates. The age of the oldest immature specimens on a body therefore yields a minimum PMI [3]. The accuracy of this estimate depends on how well field-relevant variables are captured in the underlying reference data [4,5]. The Oriental latrine fly *Chrysomya megacephala* (Fabricius, 1794) is among the most frequently recorded blow flies in forensic casework. It is often the dominant primary colonizer of human remains in tropical and subtropical Asia [6,7]. Its biology is well reviewed [8], and its thermal development is well characterized [9,10,11,12]. Less attention has been paid to the substrate into which post-feeding larvae inevitably move. Third-instar larvae disperse from the carcass and burrow into soil to pupariate [13,14]. Soil is thus an integral microenvironment of the late larval and pupal stages. Soil type and moisture significantly affect development and survival in phorid flies and *Lucilia sericata* (Meigen) [15,16]. Decomposition and sarcosaprophagous insect succession also vary with habitat conditions in tropical Malaysia [17]. However, the soils tested so far have been generic substrates. Laterite, one of the most widespread tropical soils, has never been examined as a developmental environment for forensically important flies. Laterite is a zonal soil formed under hot, humid climates through intense weathering and leaching. It is characteristically acidic, enriched in iron and aluminum (hydr)oxides and commonly in manganese, low in exchangeable bases, and clay-rich [18,19]. Laterite therefore differs from temperate reference soils in two broad respects that may each affect developing larvae: its chemistry (strong acidity and enrichment in iron and manganese (hydr)oxides) and its physical structure (a clay-rich, strongly aggregated matrix with high cohesion and moisture retention). These properties co-vary in intact laterite and are confounded in any comparison of natural soils; the present study accordingly tests the laterite effect as a whole and treats the chemical and physical components as hypotheses to be separated by future factorial experiments. Laterite covers vast tropical and subtropical areas [18,19]. In laterite-dominated regions such as much of Hainan Island, and southern China, corpses are commonly placed on, or concealed in, this soil. Larvae developing on such corpses pupariate in intimate contact with laterite. This contact is a plausible but unquantified source of variation in PMI estimation. Laterite’s metal load raises a further question. Necrophagous larvae accumulate xenobiotics, including heavy metals, which can alter developmental rates [20,21,22]. Malathion was detected in *C. megacephala* larval tissues and disturbed development [23]. Cadmium and thallium bioaccumulate in *L. sericata* larvae and puparial cases [24]. Metal transfer along a carrion food chain was quantified in the congeneric *Chrysomya albiceps* (Wiedemann, 1819) [25]. Whether chronic larval contact with an acidic, metal-rich laterite similarly perturbs growth and pupariation is unknown. Untargeted metabolomics based on ultra-high-performance liquid chromatography–tandem mass spectrometry (UHPLC-MS/MS) offers a systems-level readout of organismal responses to environmental stressors [26,27]. It is increasingly explored in forensic science, including PMI research [28,29,30]. Yet metabolome-level data on how soil environments reshape carrion-feeding larvae are essentially absent. In addition, single-site studies cannot distinguish soil-type-robust effects from site idiosyncrasies. In this preliminary exploratory study, we combined larval phenotyping (growth, pupariation timing, wandering depth), ICP-MS-based metal profiling of soil and larvae, and UHPLC-MS/MS untargeted metabolomics of whole larvae sampled on Days 1–3 post-burrowing. We compared three Hainan Island laterite soils (Sanjiang, Guanlanhu, and Chengmai; analyzed jointly as the laterite group) with a yellow earth control. A no-soil group served as a reference for soil presence itself. The design is deliberately exploratory. The primary metabolome analysis asks whether substrate-group metabolomic differences exist at all. It pools the three sampling days per soil to maximize replication (*n* = 5–6 per group). Day-resolved comparisons are reported as hypothesis-generating supplements. We tested whether laterite exposure alters larval developmental timing and whole-body metal content. Our aim was to provide baseline data for PMI inference in laterite-dominated tropical regions.

## 2. Materials and Methods

### 2.1. Soil Collection and Characterization

Three laterite soils and one yellow earth (huangtu) control soil were collected on Hainan Island, China, in a single sampling campaign. Laterite came from Sanjiang Town, Haikou (19°50′28.06″ N, 110°36′26.41″ E), Guanlanhu, Haikou (19°55′31″ N, 110°20′30.12″ E), and Chengmai County (19°28′46″ N, 109°53′5.16″ E). The yellow earth control came from Chengxi Town, Haikou (19°58′53.23″ N, 110°19′57.73″ E). The three laterite sites belong to the same laterite soil type (same parent material and soil-forming conditions). They were statistically indistinguishable and are treated as one factor (the laterite group) throughout. At each site, topsoil (0–10 cm) was collected from five points within a homogeneous plot. Debris was removed, and the soil was passed through a 2 mm sieve. Four independent soil samples per site were processed and analyzed separately (*n* = 4). The reported variation therefore reflects sample-level reproducibility within the plot rather than repeated measurement of one composite. Soil type was assigned according to the Chinese Soil Taxonomy. Soil pH was determined potentiometrically in a 1:2.5 (*w*/*v*) soil-to-water suspension (5 g air-dry, 2 mm-sieved soil) after 30 min shaking, following the Chinese national standard HJ 962-2018 (Soil—Determination of pH—Potentiometric method), using a PHSJ-3F pH meter (Shanghai INESA Scientific Instrument Co., Ltd., Shanghai, China). Each sample was measured in triplicate and averaged. Exchangeable acidity and exchangeable aluminum were not measured; total soil aluminum was quantified by ICP-MS (Section 2.5). Exchangeable Al rather than total Al is the biologically active fraction in acid soils; therefore, the absence of exchangeable-acidity data is acknowledged as a limitation (Section 4.6).

### 2.2. Fly Trapping, Species Identification, and Colony Maintenance

Adult *C. megacephala* were trapped with pig heart bait over three days on open ground at Hainan Medical University, Haikou (19°58′53.55″ N, 110°19′57.78″ E). Species identity was confirmed by morphological identification and by cytochrome c oxidase subunit I (COI) DNA barcoding against the International Nucleotide Sequence Database (INSD, including Japan DDBJ, Europe EMBL, and USA GenBank) and the National Center for Biotechnology Information (NCBI) database. Genomic DNA was extracted with the DNeasy Blood & Tissue Kit (Qiagen, Hilden, Germany; 56 °C lysis 1.5 h, spin-column purification). The COI fragment was amplified with the standard primers LCO1490 and HCO2198 in a 40 μL reaction with MonAmp™ 2×Taq Mix Pro (94 °C, 5 min; 35 cycles of 94 °C 30 s, 50 °C 45 s, 72 °C 50 s; 72 °C 10 min). Amplicons were checked by 2% agarose gel electrophoresis and bidirectionally Sanger-sequenced (BigDye Terminator v3.1, ABI 3730XL; Zhongmei Taihe Biotechnology Co., Ltd., Beijing, China). Chromatograms were proofread and assembled in SnapGene 7.3.2 after trimming primers and low-quality bases (Q < 20). Sequences with ≥98% similarity and 100% coverage in GenBank Basic Local Alignment Search Tool (BLAST + 2.17.0) searches confirmed a pure *C. megacephala* colony. Adults were maintained at 29 °C, 80% relative humidity (RH), and a 12L:12D photoperiod, with distilled water, fresh pork, and moistened wheat bran ad libitum.

### 2.3. Experimental Design and Rearing Conditions

**Figure 1:**
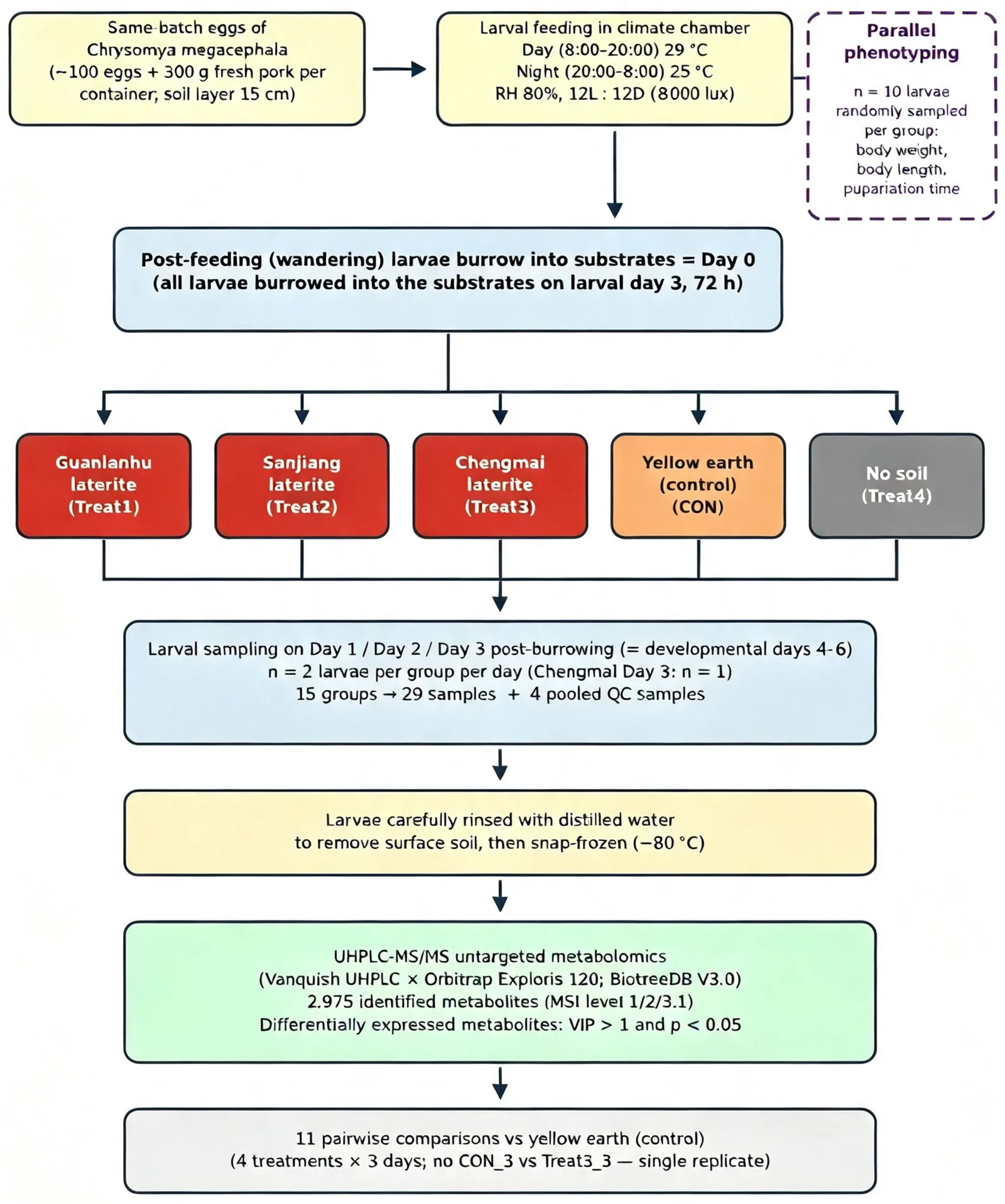
Schematic of the experimental design. Eggs (~100 per container) from a single larval batch were placed on 300 g of fresh pork over a 15 cm layer of one of three laterite soils (Treat1, Guanlanhu; Treat2, Sanjiang; Treat3, Chengmai; laterite group, pH 4.17–4.32), a yellow earth control (CON, pH 7.45), or no soil (Treat4). Larvae were reared at 29 °C day/25 °C night, 80% RH, 12L:12D, and 8000 lux. All larvae completed feeding outside the soil under identical conditions and burrowed into the substrate on their own at developmental day 3 (72 h; Day 0 post-burrowing). Days 1–3 post-burrowing therefore correspond to developmental days 4–6. Whole larvae were carefully rinsed (twice with phosphate-buffered saline before morphometry; with ultrapure water before metal analysis; with distilled water before metabolomics sampling). They were sampled on Days 1–3 post-burrowing (*n* = 2 per group per day; Treat3 Day 3, *n* = 1) for ultra-high-performance liquid chromatography–tandem mass spectrometry (UHPLC-MS/MS)-based untargeted metabolomics (29 samples in 15 groups plus 4 pooled quality controls; 2975 Metabolomics Standards Initiative level 1/2/3.1 metabolites analyzed).

The experiment comprised five groups representing four substrate conditions. Three laterite soils (Treat1, Guanlanhu; Treat2, Sanjiang; Treat3, Chengmai) jointly formed the laterite group. The yellow earth control (CON) and a no-soil group (Treat4) completed the design, with Treat4 serving as a reference for soil presence itself. All experiments used the same larval batch and soil collections. Rearing containers were filled with a 15 cm soil layer (none in Treat4). Then 300 g of fresh pork was placed on the surface, and approximately 100 eggs (variation ~10) were introduced per container. Each treatment group was reared in a single container (five containers in total). The rearing container is the experimental unit for substrate-level inference; larvae sampled from the same container share a common microenvironment and are not fully independent. Consequently, container and treatment are fully confounded in this design, and container-level effects (pseudoreplication) cannot be excluded; all group differences are therefore interpreted as preliminary (Section 4.6). All larvae completed feeding and left the food on developmental day 3 (72 h), burrowing into the substrate on their own. All larvae had developed outside the soil under identical conditions until then, so the groups were developmentally synchronized at burrowing. The burrowing day was defined as Day 0 post-burrowing, so Days 1–3 post-burrowing correspond to developmental days 4–6. The two timelines are directly aligned. All containers were kept in a climate chamber (LRH-400-GSIE) programmed identically: 29 °C day (8:00–20:00)/25 °C night (20:00–8:00), 80% RH, 12L:12D photoperiod at 8000 lux. Phenotypic and larval metal data were recorded per substrate group and reported per site, with merged laterite-group analyses where indicated. Metabolomic data were analyzed per substrate group. Figure 1 provides an overview of the workflow.

### 2.4. Phenotypic Measurements and Total Protein

Immediately after collection, larvae were rinsed twice with pre-chilled phosphate-buffered saline (PBS) to remove surface soil and food lipids and then blotted dry. To measure body length, rinsed larvae were anesthetized on ice for 5 min and photographed under a stereomicroscope (PUDA300C). Length was measured in ImageJ 1.54t (National Institutes of Health, Bethesda, MD, USA) as the straight-line distance from the mouthparts to the posterior abdominal end (calibrated scale, 0.1 mm minimum division). At each of seven observation time points (22, 34, 58, 82, 106, 130, and 154 h of developmental age), ten larvae per group were randomly sampled for body length and fresh weight, and values were averaged per group. Individual fresh weight was determined on an analytical balance (JF1004; 0.1 mg precision). Larvae were then snap-frozen in liquid nitrogen and stored at −80 °C. Pupariation events (cryptocephalic-stage onset, pupariation time) and wandering depth in the soil column were recorded by observation. Growth curves were evaluated with linear regression models. Between-group differences at each time point were tested in GraphPad Prism version 8.0.0 for Windows (GraphPad Software, San Diego, CA, USA), which was also used to prepare box plots and dual-*y*-axis line charts. Total protein was quantified with the bicinchoninic acid (BCA) assay from pooled larval homogenates (3 larvae, ~300 mg per replicate; Wuhan Servicebio Technology Co., Ltd., Wuhan, China) and expressed as extract concentration (μg/μL).

### 2.5. Metal Analysis by ICP-MS and Bioaccumulation Factors

Soil samples (0.1000–0.1009 g; four per site) were microwave-digested, brought to 10 mL, diluted 10–1000-fold as required, and analyzed by inductively coupled plasma mass spectrometry (ICP-MS). Calibration curves had R^2^ ≥ 0.9999. Larvae were analyzed by pooling three individuals per sample (~300 mg fresh weight), and four replicates per group were measured (three parallel experiments plus one independent repeat). Before digestion, larvae were rinsed with ultrapure water to remove surface-adhered soil. They were then microwave-digested as above and analyzed by ICP-MS (Wuhan Servicebio Technology Co., Ltd., Wuhan, China; instrument model not itemized in the service report). No gut evacuation (depuration) was applied in order to protect the synchrony of the parallel metabolomic and phenotypic measurements: depuration would have required transferring soil-exposed larvae to a soil-free substrate for several hours, which would have altered their physiological state within the same 96–144 h post-burrowing window used for metabolomics and phenotyping and decoupled the measurements. Larval values therefore represent whole larval content, including gut contents, while rinsing excluded surface contamination. Consequently, the ICP-MS results cannot distinguish tissue-incorporated metals from ingested soil particles retained in the gut: the larval Fe and Mn elevations should be read as upper bounds of tissue uptake rather than as bioaccumulation into larval tissues, and the bioaccumulation factors are approximations likewise affected by retained gut soil. Quantified elements were Fe, Mn, Zn, Ca, Mg, K, and Al. The bioaccumulation factor (BAF) was defined as larval concentration divided by soil concentration (both μg/g, dry weight; BAF > 1 indicates accumulation). It was computed from per-sample concentrations and averaged. Larvae were not dried, and water content was not measured, so the dry-weight-based BAF values are approximations. Larval metal content and BAFs are reported per substrate (*n* = 4 per group). The three laterites did not differ detectably in any element (one-way ANOVA); therefore, laterite-versus-control conclusions were confirmed with merged tests (*n* = 12) alongside per-site tests.

### 2.6. Metabolomics Sampling and Grouping

For metabolomics, whole larvae were collected from the same cohort on Days 1, 2, and 3 post-burrowing (24, 48, and 72 h), rinsed with distilled water, snap-frozen in liquid nitrogen, and stored at −80 °C. Each group-by-day combination comprised two biological replicates (*n* = 2), except the Chengmai laterite on Day 3 (Treat3_3, one valid sample). This gave 29 larval samples in 15 groups plus 4 pooled quality control (QC) samples. Untargeted profiling was performed by Shanghai BIOTREE Biological Technology Co., Ltd. (Shanghai, China).

### 2.7. Metabolite Extraction

Larval tissue (25 mg) was mixed with two homogenization beads and 500 μL of extraction solvent (methanol:acetonitrile:water, 2:2:1, *v*/*v*/*v*) containing isotope-labeled internal standards, vortexed (30 s), homogenized (35 Hz, 4 min), and sonicated in an ice-water bath (5 min) three times. After protein precipitation at −40 °C for 1 h, 300 μL of supernatant was filtered through a 96-well filtration plate under positive pressure (6 psi, 3 min), and the filtrate was analyzed. Equal aliquots of all samples were pooled to generate QC samples.

### 2.8. UHPLC-MS/MS Analysis

Chromatographic separation used a Vanquish UHPLC system (Thermo Fisher Scientific, Waltham, MA, USA) with a Waters ACQUITY UPLC BEH Amide column (2.1 mm × 50 mm, 1.7 μm). The mobile phases were (A) 25 mmol/L ammonium acetate and 25 mmol/L ammonium hydroxide in water and (B) acetonitrile. The autosampler was held at 4 °C, and the injection volume was 2 μL. Mass spectrometry used an Orbitrap Exploris 120 (Thermo Fisher Scientific) controlled by Xcalibur v4.4 in positive (+3.8 kV) and negative (−3.4 kV) electrospray modes. The full-MS resolution was 60,000 and the MS/MS resolution was 15,000. Other settings were stepped normalized collision energies (SNCE) 20/30/40, sheath and auxiliary gas 50 and 15 Arb (arbitrary units), and capillary 320 °C. The chromatographic gradient program was not itemized in the service report. The standard m/z scan range of the Thermo Orbitrap Exploris 120 (40–3000) was also not listed.

### 2.9. Data Processing, Metabolite Identification, and Statistical Analysis

Raw files were converted to mzXML with ProteoWizard (v3.0.24054) and processed with the XCMS-based R pipeline. Features were denoised by relative standard deviation (RSD) filtering. Features missing in >50% of samples in any group were removed; remaining missing values were imputed with half the minimum, and data were normalized by total ion current (TIC). Of 81,507 extracted features, 81,501 were retained. From these, 2975 metabolites with MS/MS-level identification (Metabolomics Standards Initiative, MSI, levels 1, 2, and 3.1: 609, 2122, and 244) entered differential analysis. They were annotated with BiotreeDB (V3.0) under the standard MSI scheme (level 1, authentic-standard match; level 2, library match; level 3, theoretical-library match; level 4, unknown). The primary differential analysis was performed by the authors from the normalized matrix using Python 3 (scipy, statsmodels, scikit-learn). In each treatment, the three sampling days were pooled (CON vs. Treat1, Treat2, Treat4, *n* = 6; Treat3, *n* = 5). Each of the 2975 metabolites was tested with a two-sided Student’s *t*-test on normalized intensities, followed by Benjamini–Hochberg false discovery rate (FDR) correction across all tests (q < 0.05). Fold change (FC) was defined as treatment/CON (log2FC > 0, higher in treatment). In each comparison, a one-component partial least squares–discriminant analysis (PLS-DA) was fitted on log10-transformed, row-centered intensities. This yielded variable importance in projection (VIP) scores and leave-one-out cross-validation (LOO-CV) performance (Q2, R2Y). Merged-analysis differentially expressed metabolites (DEMs) were defined by VIP > 1 and unadjusted *p* < 0.05 (≈149 of 2975 tests expected to be significant by chance at α = 0.05). Day-wise comparisons (*n* = 2 per group-day; Table S4) were re-analyzed with the same procedure. The day-resolved results are hypothesis-generating supplements. Per group-day (*n* = 2), orthogonal partial least squares–discriminant analysis (OPLS-DA) models were fitted in SIMCA v18.0.1 (Sartorius) after log transformation and unit-variance scaling (7-fold cross-validation, 200 permutations). Day-wise DEMs were defined by VIP > 1 (first predictive component) and Student’s *t*-test *p* < 0.05 without FDR correction. CON_3 vs. Treat3_3 was impossible (one sample). Cross-laterite shared DEMs per day were recomputed from the per-comparison DEM lists with feature-level deduplication (one entry per unique m/z–retention-time pair). The vendor Venn matrix (0/1 membership of the 1216 union DEMs across the 11 comparisons) undercounted the intersections because some DEM names were absent from its union list; the reconciled counts (5, 15, and 69 shared DEMs on Days 1–3) are documented in Table S8. Pathway analysis used the Kyoto Encyclopedia of Genes and Genomes (KEGG) with *Drosophila melanogaster* as a reference. Enrichment used Fisher’s exact test (Rich Factor), and the differential abundance (DA) score was (up − down)/total annotated metabolites per pathway. Gene set enrichment analysis (GSEA) and MetaboAnalyst topology analysis were complementary.

### 2.10. Statistical Analysis of Phenotypic and Metal Data

Phenotypic and metal data are reported as mean ± SD. Between-group comparisons used independent-samples *t*-tests (each laterite group and the merged laterite group, *n* = 12, vs. the control). Homogeneity among the three laterites was assessed with one-way analysis of variance (ANOVA), and growth curves were evaluated with linear regression models. Analyses and plotting used GraphPad Prism, with *p* < 0.05 considered significant.

## 3. Results

### 3.1. Soil Physicochemical Properties and Metal Content

**Figure 2:**
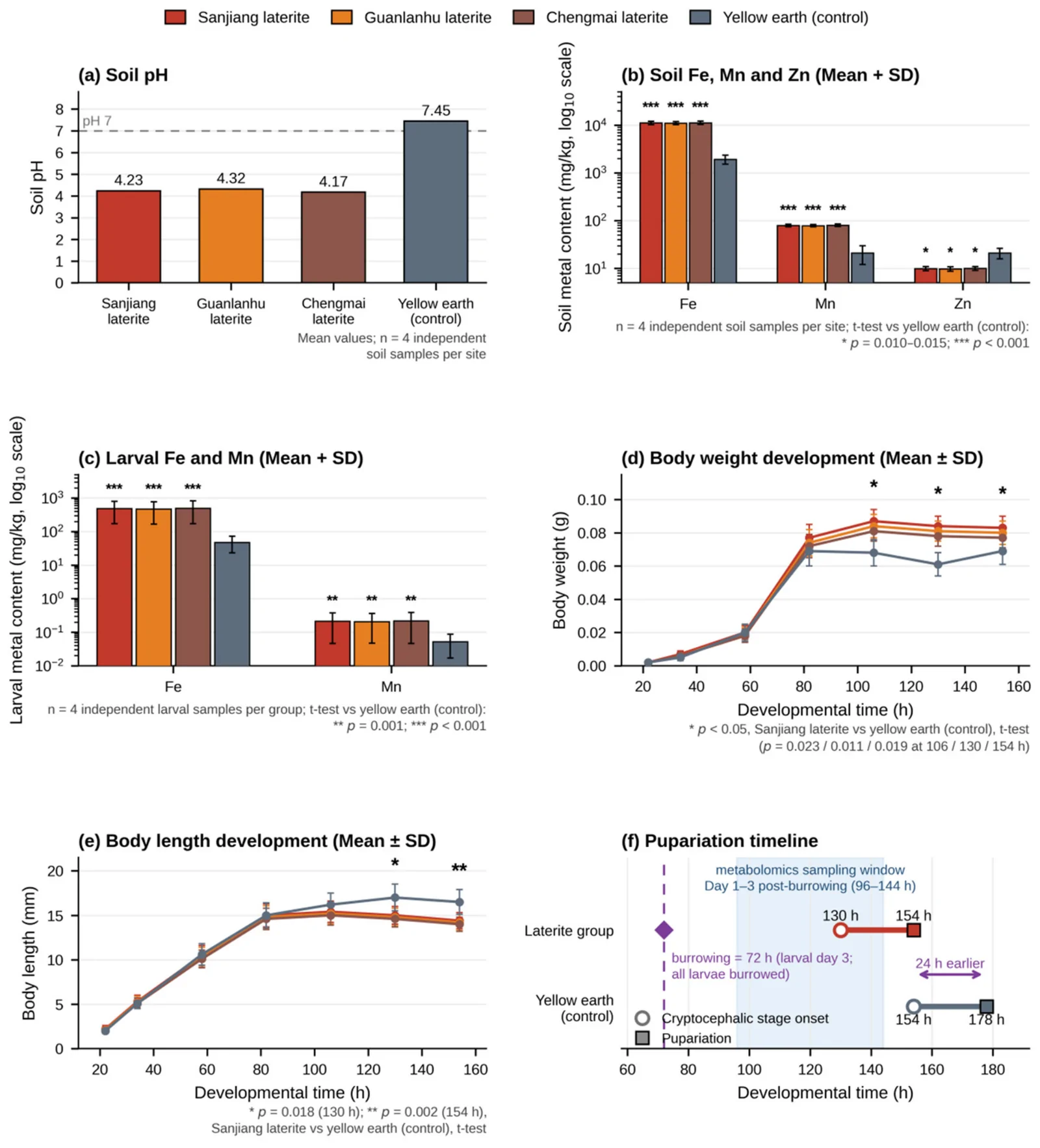
Soil characteristics, whole-larval metal content, and developmental phenotypes of the four groups (three laterites—Sanjiang, Guanlanhu, Chengmai—and the yellow earth control). (**a**) Soil pH (Sanjiang 4.23, Guanlanhu 4.32, Chengmai 4.17, control 7.45). (**b**) Soil Fe, Mn, and Zn content of the four soils (mg/kg, log10 *y*-axis; mean + SD, *n* = 4 independent soil samples per site; *** *p* < 0.001, * *p* < 0.05, independent-samples *t*-test, each laterite vs. yellow earth). (**c**) Whole-larval Fe and Mn content of the four groups (mg/kg, log10 *y*-axis; mean + SD, *n* = 4 per group; *** *p* < 0.001, ** *p* = 0.001, each laterite vs. control); larvae were rinsed before digestion (no gut evacuation), but values still include gut contents. (**d**) Body weight over developmental time (mean ± SD, *n* = 10 larvae per group per time point; seven time points, 22–154 h); laterite groups were significantly heavier than the control from 106 h through 154 h (* *p* < 0.05, per-time-point *t*-tests). (**e**) Body length over developmental time (mean ± SD, *n* = 10; same seven time points); laterite groups were significantly shorter at 130 and 154 h (* *p* < 0.05, ** *p* < 0.01; symbols denote the Sanjiang vs. control comparison; all three laterite groups were significant at both time points, Table S7b); note the length peak at 106 h followed by a decline (earlier post-feeding contraction) in the laterite groups versus continued elongation to 130 h in the control. (**f**) Developmental timelines of the laterite group and the control on a shared developmental-age axis (hours): onset of the cryptocephalic stage (130 vs. 154 h; ≈Day 2.4 vs. ≈Day 3.4 post-burrowing) and pupariation (154 vs. 178 h; 24 h advance), with burrowing at 72 h marked and the post-burrowing timeline indicated by the shaded metabolomics sampling window (Days 1–3 post-burrowing = 96–144 h, developmental days 4–6).

The three laterites were strongly acidic (pH 4.17–4.32), and the yellow earth control was near-neutral (pH 7.45). The laterites were statistically indistinguishable in metal content at the available replication (one-way ANOVA, *p* = 0.952–0.998, *n* = 4 per site; Table 1, Figure 2a,b). Iron and manganese were significantly enriched in every laterite (Fe ~5.7–5.8-fold, Mn ~3.7–3.8-fold; *p* < 0.001 each), whereas zinc was significantly lower (*p* = 0.013–0.015). Calcium, magnesium, aluminum, and potassium did not differ (*p* > 0.05). The *n* = 4 samples per site were independent; generalization beyond the plots is addressed.

### 3.2. Larval Metal Content and Bioaccumulation Factors

Whole-larval metal content differed markedly between groups for the two dominant soil metals (Table 2, Figure 2c). Values were quantified after ultrapure-water rinsing, which excluded surface soil but not gut contents. The three laterites were statistically indistinguishable for every element at the available replication (one-way ANOVA, *p* = 0.987–0.997). Conclusions are therefore reported from per-site tests (*n* = 4) and confirmed in merged laterite-group tests (*n* = 12). Whole-larval Fe (including gut contents) reached 489.5 ± 317.2, 472.8 ± 305.6, and 501.2 ± 328.9 mg/kg (Sanjiang, Guanlanhu, Chengmai) vs. 48.1 ± 24.8 mg/kg in controls (each *p* < 0.001; merged t = 8.51; ~10.2-fold). Whole-larval Mn (including gut contents) reached 0.212 ± 0.166, 0.205 ± 0.158, and 0.218 ± 0.172 vs. 0.052 ± 0.035 mg/kg (each *p* = 0.001; merged *p* < 0.001; ~4.1-fold). As rinsing excluded surface contamination, this elevation reflects metals within the larvae. However, tissue uptake cannot be separated from gut contents. Whole-larval Ca was significantly lower in every laterite group (0.18 ± 0.24, 0.17 ± 0.22, and 0.19 ± 0.26 vs. 1.09 ± 0.93 g/kg; each *p* = 0.001; merged t = −4.98). In contrast, Zn (0.153 ± 0.111, 0.148 ± 0.105, and 0.158 ± 0.118 vs. 0.140 ± 0.055 mg/kg; merged t = 0.33, *p* = 0.743) and Mg (1.01 ± 0.75, 0.98 ± 0.72, and 1.04 ± 0.78 vs. 0.89 ± 0.39 g/kg; merged t = 0.45, *p* = 0.654) did not differ. Aluminum was below the limit of detection (<0.01 mg/kg), with low detection rates and large fluctuation, and was not analyzed. Potassium was excluded as physiologically implausible (Table 2 note). BAF values indicated no biomagnification of Fe, Mn, or Zn. All three had BAF < 1 in both groups (merged laterite vs. control: Fe 0.04 vs. 0.03; Mn 0.003 vs. 0.003; Zn 0.02 vs. 0.01). Calcium showed BAF > 1 only in the laterite group (2.56 vs. 0.73), and magnesium showed BAF > 1 in both groups (12.6 vs. 4.9). However, larval Mg did not differ between groups, and larval Ca was lower in the laterite group. These essential-element BAFs therefore reflect physiological regulation, not biomagnification.

### 3.3. Larval Growth, Development, and Pupariation Phenotype

Larvae raised on each laterite developed a distinct “heavier but shorter” phenotype in the late developmental window (complete series in Table S7a–c; Figure 2d,e). Body weight did not differ among the four groups through 82 h (22 h: 0.002 ± 0.001 g in all groups; 34 h: 0.007, 0.006, and 0.006 vs. 0.005 ± 0.002 g; 58 h: 0.020, 0.019, and 0.018 vs. 0.020 ± 0.005 g; 82 h: 0.077, 0.074, and 0.072 vs. 0.069 ± 0.009 g; Sanjiang, Guanlanhu, and Chengmai vs. control; all *p* > 0.05; Figure 2d). From 106 h (developmental day 4.4) through 154 h, however, body weight was significantly higher in all laterite groups: at 106 h, 0.087 ± 0.007, 0.084 ± 0.007, and 0.081 ± 0.006 vs. 0.068 ± 0.008 g (*p* = 0.023, 0.031, and 0.042); at 130 h, 0.084, 0.081, and 0.078 vs. 0.061 ± 0.007 g (*p* = 0.011, 0.018, and 0.029); and at 154 h, 0.083, 0.080, and 0.077 vs. 0.069 ± 0.008 g (*p* = 0.019, 0.027, and 0.038). Body length likewise did not differ through 106 h (22 h: 2.2, 2.1, and 2.0 vs. 2.0 mm; 82 h: 15.0, 14.8, and 14.6 vs. 15.0 mm; 106 h: 15.4, 15.2, and 15.0 vs. 16.2 ± 1.3 mm; all *p* > 0.05; Figure 2e). Laterite larvae were significantly shorter at 130 h (15.0 ± 1.0, 14.8, and 14.6 vs. 17.0 ± 1.5 mm; *p* = 0.018, 0.012, and 0.007) and 154 h (14.4 ± 0.9, 14.2, and 14.0 vs. 16.5 ± 1.4 mm; *p* = 0.002, 0.001, and <0.001). No group differences in body length were detected at 22, 34, 58, 82, or 106 h (all *p* > 0.05). Laterite-group body length peaked at 106 h and declined thereafter (Sanjiang, 15.4 → 15.0 → 14.4 mm), whereas controls elongated until 130 h (16.2 → 17.0 mm). This indicates an earlier onset of post-feeding contraction, consistent with the earlier cryptocephalic stage (below). Each time point was an independent random sample (*n* = 10 per group); consequently, within-group trajectories (peak and subsequent decline) are inferred from cross-sectional group means rather than longitudinal tracking of individuals. The three laterite groups did not differ from each other at any time point (ANOVA, *p* > 0.05, *n* = 10 per group), consistent with cross-site robustness. Developmental timing shifted toward earlier pupariation. The cryptocephalic stage began at 130 h vs. 154 h in controls, and pupariation occurred at 154 h vs. 178 h, that is, 24 h earlier. Wandering laterite-group larvae also concentrated in the shallow 3–5 cm layer, whereas controls migrated to 8–10 cm. In addition, soil clods formed around the pork in the laterite group. Total larval protein did not differ between groups (7.96 ± 1.44, 7.67 ± 1.21, and 7.72 ± 1.03 vs. 7.40 ± 1.45 μg/μL; all *p* > 0.05). The cryptocephalic stage began at ≈Day 2.4 post-burrowing in the laterite group. This falls inside the metabolomics window (Days 1–3 post-burrowing = developmental days 4–6), the window of the weight and length differences. In the control, the cryptocephalic stage began at ≈Day 3.4, just after this window. Pupariation occurred after the window in both groups. The window thus brackets the prepupal transition for the laterite group but not the control (Figure 2f): laterite larvae were metamorphosing while control larvae were still wandering, so developmental stage and substrate are confounded within this window. This distinction is central to interpreting the metabolome data (Section 4.3).

### 3.4. Metabolome Quality Control and Overall Profiles

**Figure 3:**
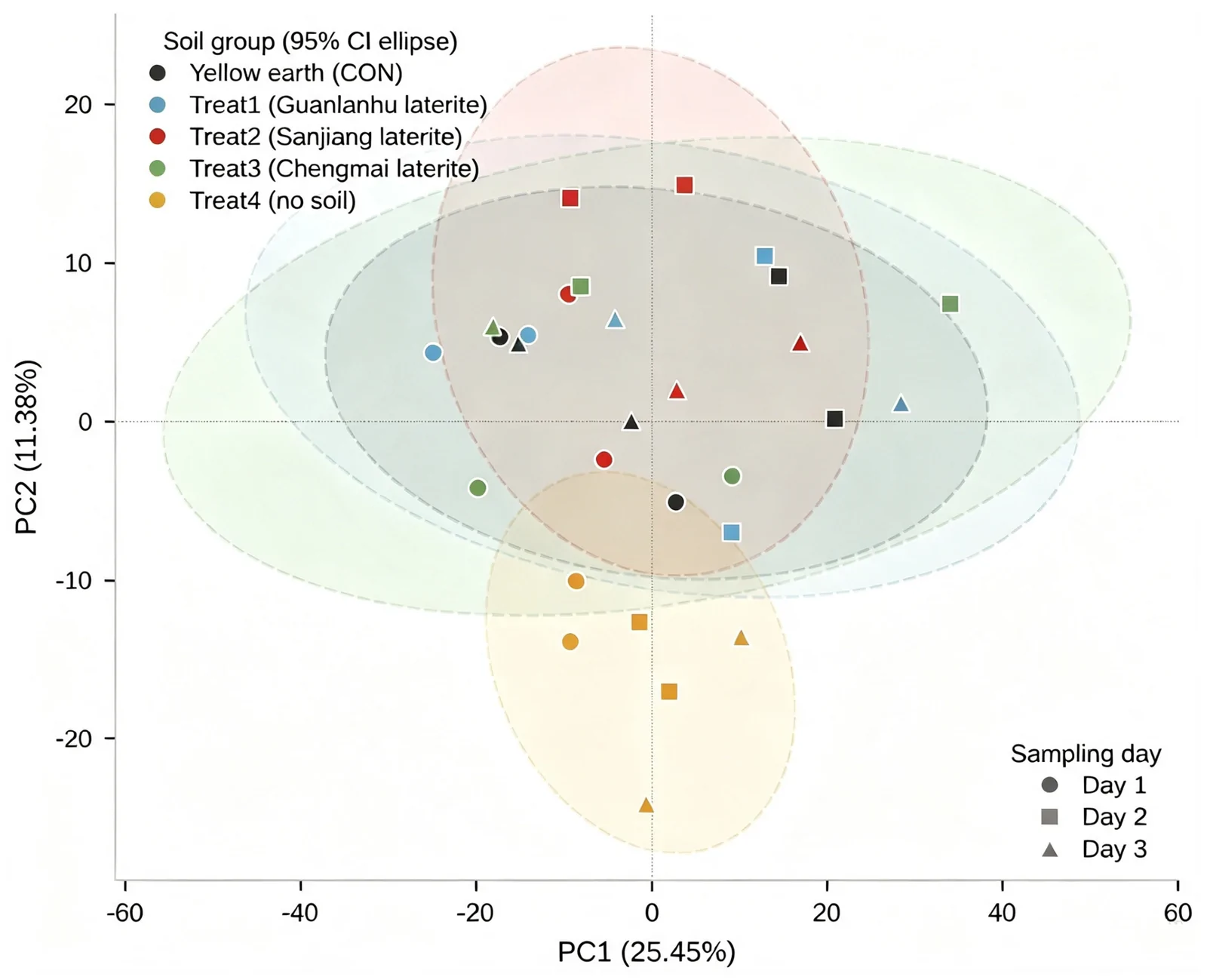
Principal component analysis (PCA) score plot of the larval metabolomes, independently recomputed from the 2975 identified metabolites (Metabolomics Standards Initiative levels 1–3.1; log10-transformed and row-centered) for all 29 samples of the five substrate groups (CON, yellow earth control; Treat1, Guanlanhu laterite; Treat2, Sanjiang laterite; Treat3, Chengmai laterite; Treat4, no soil). Points are colored by substrate group and shaped by sampling day (circle/square/triangle = Day 1/2/3); dashed ellipses are 95% confidence ellipses per substrate group across days (χ^2^, df = 2). PC1 and PC2 explain 25.45% and 11.38% of the variance, respectively. The groups overlap substantially, consistent with the limited unsupervised separation achievable at *n* = 2 per group-day and with the low predictive performance of the supervised merged models; the plot is intended for visualization only.

Of the 81,501 preprocessed features (29 larval samples), 2975 metabolites with MS/MS-level identification (MSI levels 1–3.1) entered analysis. Quality control was satisfactory. The four pooled QC samples clustered tightly in principal component analysis (PCA); all samples fell within ±2 SD in one-dimensional PCA distribution, QC correlations exceeded 0.85, and the six isotope-labeled internal standards showed RSDs of 0.18–1.51% with no appreciable blank peaks. In the unsupervised PCA, the first six components explained R2X(cum) = 0.527. A PCA recomputed from the 2975 identified metabolites gave first-two-component variances of 25.45% and 11.38%, with substantial substrate-group overlap (Figure 3). Such limited separation is expected with two replicates per group-day and 2975 variables. The day-wise OPLS-DA models all achieved group separation (R2Y ≥ 0.998, Q2 = 0.573–0.973). However, at *n* = 2 and near-unity R2Y, these models carry overfitting risk and are descriptive only (Table S4).

### 3.5. Primary Merged Differential Analysis: No FDR-Significant Laterite Metabolites, Three in the No-Soil Comparison

**Figure 4:**
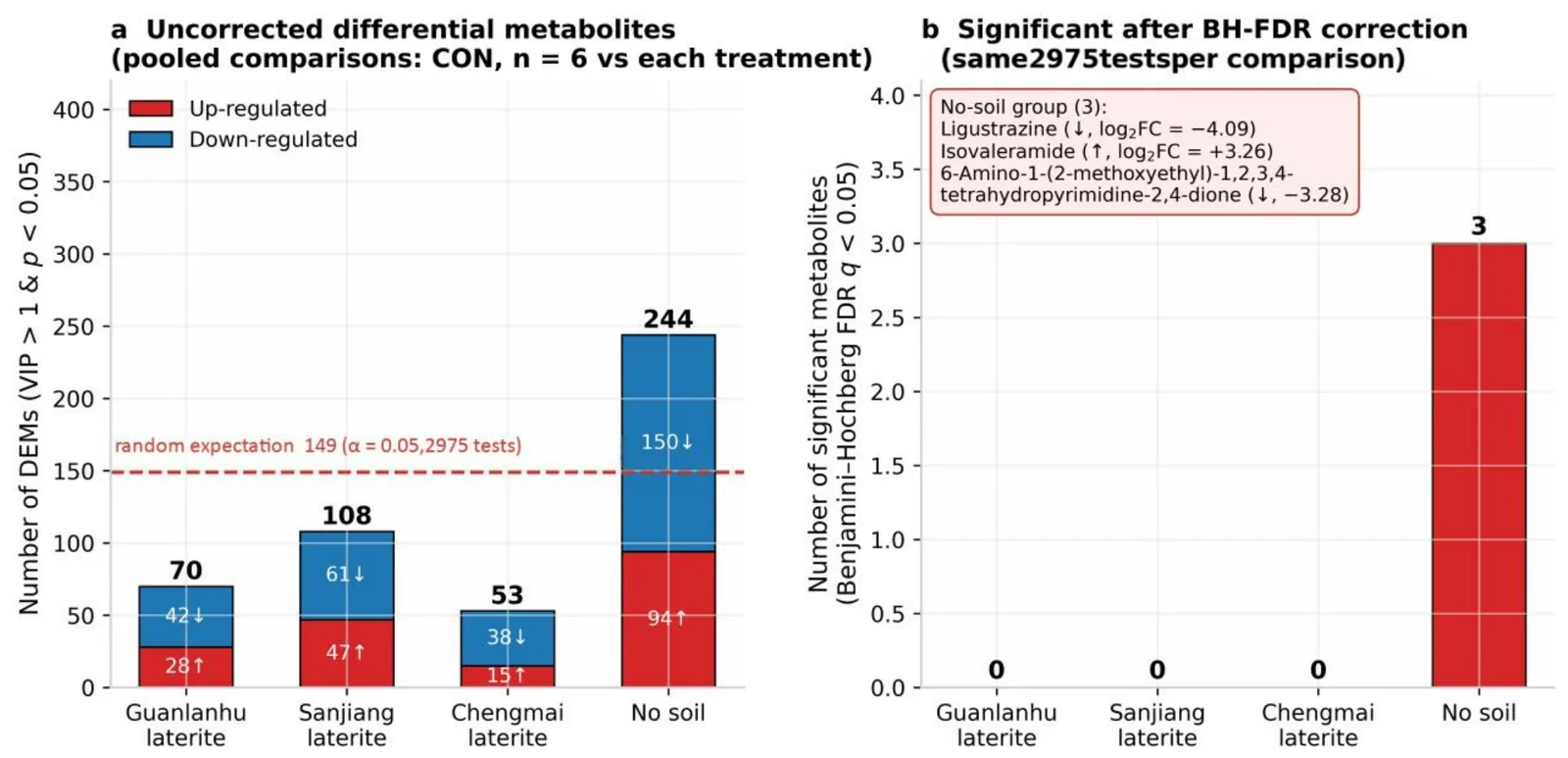
Primary merged differential analysis of the four substrate comparisons (three sampling days pooled; *n* = 6 per group, Treat3 *n* = 5; 2975 metabolites tested per comparison). (**a**) Numbers of unadjusted differentially expressed metabolites (DEMs; variable importance in projection, VIP > 1 and *p* < 0.05; red, up-regulated; blue, down-regulated relative to the yellow earth control); the dashed line marks the random expectation at α = 0.05 (≈149 of 2975 tests). (**b**) Numbers of DEMs significant after Benjamini–Hochberg correction (q < 0.05): 0 in all three laterite comparisons and 3 in the no-soil comparison.

**Figure 5:**
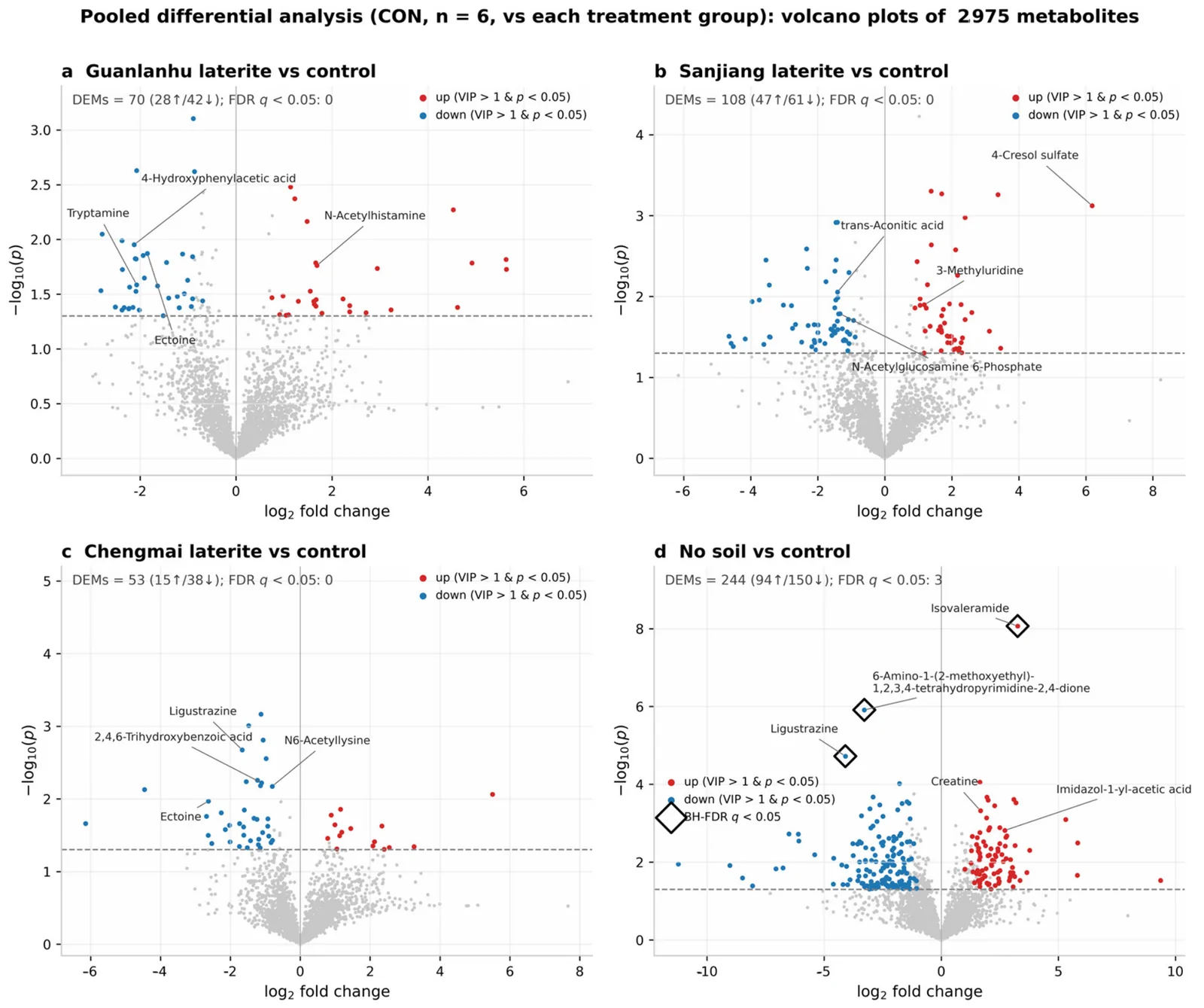
Volcano plots of the four merged comparisons (log2 fold change vs. −log10(*p* value); 2975 metabolites per comparison; three sampling days pooled). Gray points, metabolites not meeting the unadjusted criterion; colored points, unadjusted DEMs (VIP > 1 and *p* < 0.05; red, up-regulated; blue, down-regulated). Metabolomics Standards Initiative level-1 candidate metabolites are labeled; the three metabolites significant after Benjamini–Hochberg correction (no-soil comparison only, Table 4) are highlighted with black outlines.

In the merged analysis, 2975 metabolites were tested per comparison (Table 3, Figure 4). Under the unadjusted criterion (VIP > 1, *p* < 0.05), the laterite comparisons yielded 70, 108, and 53 DEMs (Treat1–3), and the no-soil comparison yielded 244 (up/down splits in Table 3). Against the random expectation (≈149 of 2975 tests at α = 0.05), only the no-soil comparison exceeded chance. After the Benjamini–Hochberg correction, no metabolite remained significant in any laterite comparison (q < 0.05: 0, 0, 0), whereas three did in the no-soil comparison (Table 4). Consistent with this, the one-component PLS-DA models of the laterite comparisons showed no predictive ability (LOO-CV Q2 ≤ 0.037), whereas the no-soil model performed well (Q2 = 0.715; Table 3). The cross-laterite intersection of the unadjusted merged DEM sets contained only two compounds, (−)-noroxymorphone and famprofazone, with inconsistent directions across laterites. Both are xenobiotic-like MSI level-2 annotations and probable false positives in a fly larva matrix (Table S3). No robust laterite-shared signal, therefore, exists at the present replication. In contrast, the no-soil group differed from the control beyond chance expectation, indicating a detectable effect of soil absence itself. Volcano plots are shown in Figure 5.

### 3.6. Candidate MSI Level-1 Metabolites in the Laterite Comparisons

We restricted interpretation to the highest-confidence identifications and extracted the MSI level-1 metabolites in the unadjusted merged DEM sets: 17, 15, and 8 compounds in the Guanlanhu, Sanjiang, and Chengmai comparisons, respectively (Table 5; complete lists in Table S5). None survived FDR correction (all q ≥ 0.45). These are directional candidates, not confirmed differences. The Sanjiang set was enriched for decreased central carbon compounds (trans-aconitic acid, fumaric acid, and pyruvaldehyde (methylglyoxal)) and for increased 4-cresol sulfate and the medium-chain acylcarnitines octanoylcarnitine and hexanoylcarnitine (statistics in Table 5). The Guanlanhu (15 of 17) and Chengmai (6 of 8) sets were likewise dominated by decreases, including ectoine in both. They are a prioritization list for targeted validation, not evidence of metabolic reprogramming.

### 3.7. Exploratory Day-Resolved Patterns and KEGG Enrichment

**Figure 6:**
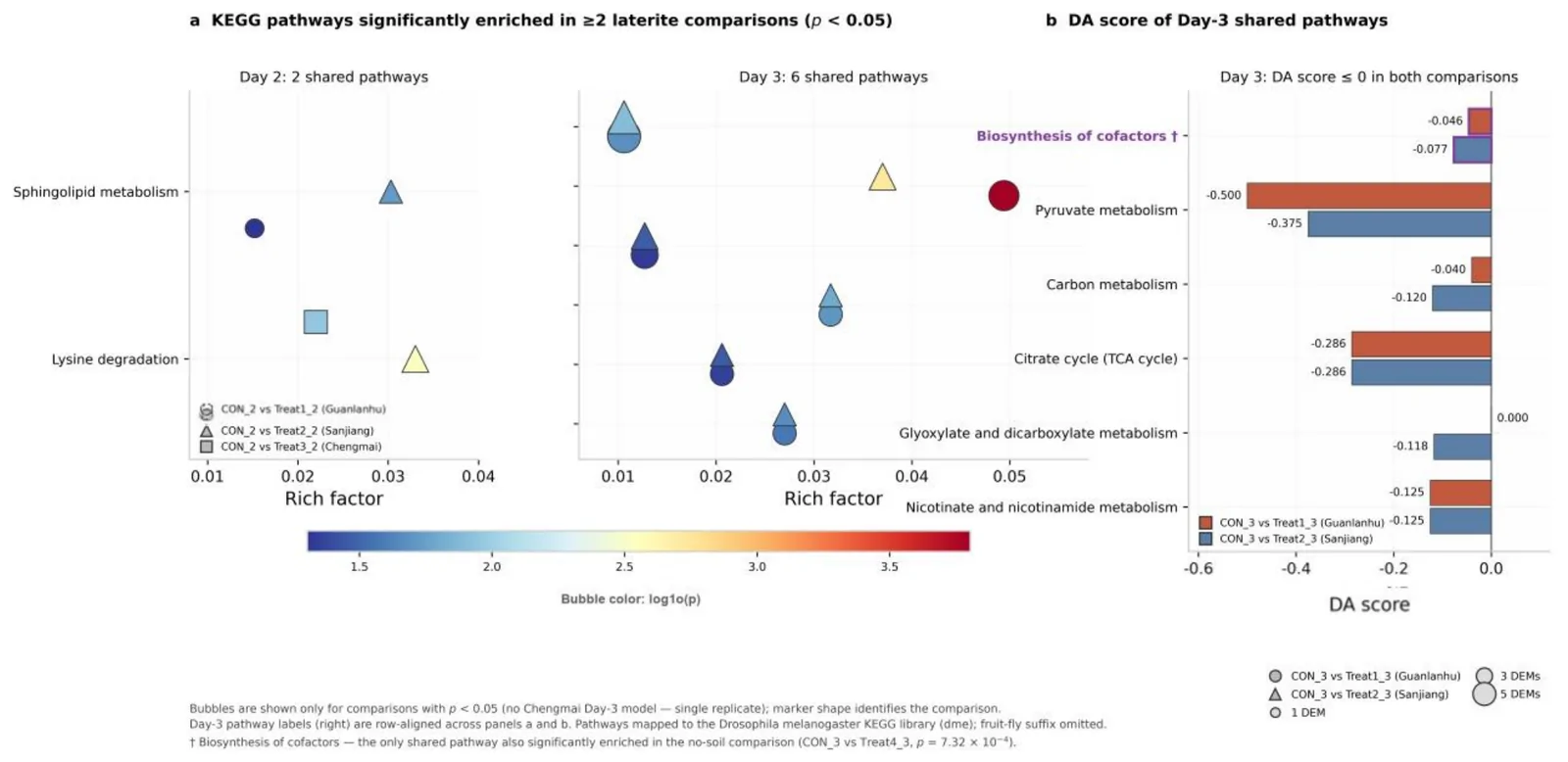
Exploratory KEGG over-representation analysis of the day-resolved differentially expressed metabolites (reference species Drosophila melanogaster; Fisher’s exact test, uncorrected; hypothesis-generating). (**a**) Dot plots of the pathways shared across the laterite comparisons: Day 3, six pathways shared by the two available comparisons (CON_3 vs. Treat1_3 and CON_3 vs. Treat2_3; *p* < 0.05 in both)—pyruvate metabolism, citrate (TCA) cycle, carbon metabolism, glyoxylate and dicarboxylate metabolism, nicotinate and nicotinamide metabolism, and biosynthesis of cofactors. Day 2: two shared pathways (sphingolipid metabolism and lysine degradation). Biosynthesis of cofactors was also significantly enriched in the Day 3 no-soil comparison (*p* = 7.32 × 10^−4^). Dot size, number of differential metabolites in the pathway; color, −log10(*p* value). (**b**) Differential abundance (DA) scores of the six shared pathways in the two comparisons (all ≤ 0, a coordinated down-regulation trend); five of the six were not significantly enriched in the Day 3 no-soil comparison. Note that the underlying day-wise DEM sets were defined without FDR correction and did not survive Benjamini–Hochberg re-analysis (Table S4), so the pathway patterns are exploratory.

The day-resolved results (*n* = 2 per group-day; no FDR correction) are hypothesis-generating patterns (Figure 6; Tables S4 and S6; Figure S1). Day-wise DEM counts varied widely (Day 1: 165, 96, 54, and 145; Day 2: 97, 248, 104, and 371; Day 3: 144, 155, not available, and 247; Treat1-4). Several counts were near the approximate 149 expectation. The authors’ Benjamini–Hochberg re-analysis found no significant day-wise metabolite except one in the Day 3 no-soil comparison (Table S4). The cross-laterite intersection, recomputed from the per-comparison DEM lists with feature-level deduplication (one entry per unique feature, i.e., per unique m/z–retention-time pair), identified 5, 15, and 69 shared DEMs on Days 1–3 (Day 3 based on two available comparisons); each shared entry corresponds to a single feature with identical m/z and retention time across comparisons. The earlier Venn-matrix counts (2, 3, and 48) undercounted the intersections because the vendor matrix omitted some DEM names from its union list; the reconciled counts are now used consistently in the text, Table S8, and Figure S1. The Day 1 shared set comprises bergenin, cyclic APT, etobenzanid, famprofazone, and sodium aluminium oxide (NaAlO2), of which famprofazone changed direction inconsistently across sites (Table S8). Of these 69 Day 3 compounds, 67 changed in the same direction across sites, and 40 of the 69 were not differentially expressed in the no-soil comparison. These shared sets include xenobiotic-like level-2 names (e.g., famprofazone, trifloxystrobin, ‘Antibiotic WB’). Such names are probable false positives in a fly larva matrix. They are listed in Tables S3–S5 and are not interpreted biologically. KEGG over-representation analysis (*Drosophila melanogaster* reference; Fisher’s exact test, uncorrected) found six pathways shared by the two available Day 3 laterite comparisons: pyruvate metabolism, the citrate (TCA) cycle, carbon metabolism, glyoxylate and dicarboxylate metabolism, nicotinate and nicotinamide metabolism, and biosynthesis of cofactors. Each had a negative or zero differential abundance (DA) score in both comparisons (−0.500 to 0.000), a coordinated down-regulation trend. Five of the six were not enriched in the no-soil comparison (Figure 6; Table S6). On Day 2, two pathways (sphingolipid metabolism and lysine degradation) were shared by the laterite comparisons (Figure 6a). The complementary analyses did not pass FDR correction. GSEA returned one significant set across all 11 day-wise comparisons (arachidonic acid metabolism, Day 3 no-soil; normalized enrichment score (NES) = 2.12, adjusted *p* = 0.026). MetaboAnalyst topology analysis returned none. The underlying DEMs are not FDR-controlled; consequently, these patterns only prioritize hypotheses.

## 4. Discussion

### 4.1. Laterite as a Developmental Microenvironment: A “Heavier, Shorter, and Earlier” Phenotype

Tropical laterite altered both the morphology and the schedule of *C. megacephala* development. Larvae became significantly heavier from 106 h and significantly shorter from 130 h. They entered the cryptocephalic stage at 130 vs. 154 h, pupariated 24 h earlier (154 vs. 178 h), and pupariated in shallower soil (3–5 vs. 8–10 cm). The three laterites were physicochemically indistinguishable at the available replication. They also induced concordant phenotypes across sites throughout the seven-time-point series. The syndrome therefore reflects soil type rather than one idiosyncratic site, although confirmation at further sites is needed. To our knowledge, this is the first report of a laterite effect on any forensically important fly. It extends earlier work on generic soils [15,16].

Two non-exclusive driver groups may explain this syndrome. The first is physical. Laterite is a clay-rich, strongly aggregated soil [18,19]. It formed clods around the food, and its fine, cohesive matrix plausibly impeded burrowing. Larvae arrested their descent at 3–5 cm rather than migrating to 8–10 cm as in the loose yellow earth. Dispersal and burial are integral to post-feeding behavior [13,14]; therefore, truncated burrowing may truncate the wandering phase itself and bring pupariation forward. The “heavier but shorter” shape suggests that growth was decoupled from longitudinal extension. Adhering soil cannot explain the greater weight because larvae were rinsed twice with PBS. Retained gut contents (no depuration) could nonetheless have contributed. The resolved length trajectories document a coherent morphological chain. Laterite larvae ceased elongating after 106 h and then shortened (Sanjiang, 15.4 → 15.0 → 14.4 mm), while controls grew to 130 h (16.2 → 17.0 mm). This pattern indicates earlier entry into the post-feeding contraction phase. It matches the earlier cryptocephalic stage onset (130 vs. 154 h) and the 24 h earlier pupariation. The shorter length on days 5–6 thus largely reflects this stage shift rather than impaired elongation per se, although a genuine growth component cannot be excluded.

The second driver group is chemical. The laterites were strongly acidic (pH 4.17–4.32 vs. 7.45) and Fe/Mn-enriched (~5.7–5.8- and ~3.7–3.8-fold). Chronic contact with such a matrix is a plausible stressor. All larvae burrowed at the same age (72 h), and the phenotypic differences therefore coincide with the metabolomics window. This window brackets the cryptocephalic transition only in the laterite group (approx. Day 2.4 vs. approx. Day 3.4). Whether the acceleration is adaptive (early escape from an unfavorable substrate) or pathological (stress-induced premature metamorphosis) cannot be decided with the present data.

### 4.2. Elevated Whole-Larval Iron and Manganese Without Biomagnification

Whole-larval iron and manganese exceeded the control by ~10.2- and ~4.1-fold in each laterite group. The three laterite groups were mutually indistinguishable for every element (one-way ANOVA, *p* = 0.987–0.997). The conclusions are robust to site choice, as the merged tests (*n* = 12) rest on three independent sites. Rinsing excluded surface-adhered particles. However, without gut evacuation the values include gut contents and cannot be equated with tissue uptake. Such elevation is consistent with entomotoxicological evidence that necrophagous larvae take up metals from their surroundings [20,21,22,23,24,25], although the congeneric *Chrysomya albiceps* may handle metals differently. The BAF values bound the interpretation. For Fe, Mn, and Zn, BAF remained far below 1 in both groups (Fe 0.04 vs. 0.03; Mn 0.003 vs. 0.003; Zn 0.02 vs. 0.01). The higher whole-larval contents therefore track soil concentrations rather than active accumulation. Zinc homeostasis appeared robust: despite significantly lower soil Zn, larval Zn did not differ. Magnesium likewise did not differ (merged *p* = 0.654), despite BAF > 1 in both groups (12.6 vs. 4.9). This is consistent with tight physiological regulation of an essential element. Calcium showed BAF > 1 exclusively in the laterite group (2.56 vs. 0.73), even though larval Ca was lower there (0.18 vs. 1.09 g/kg). In an acidic, base-poor soil, upregulated uptake is a plausible compensatory response. This agrees with the strong element- and matrix-dependence of BAF in blow fly larvae [24]. All BAF values (dry-weight basis) are approximations.

### 4.3. The Larval Metabolome: A Detectable Soil-Presence Effect but No Robust Laterite-Specific Signal

The merged analysis answers the primary question, whether substrate groups differ metabolically at all, with maximal replication (*n* = 5–6 per group). All three laterite comparisons showed unadjusted DEM counts below chance expectation, no FDR-significant metabolite, no predictive supervised models, and only two probable false positives as cross-laterite shared candidates. Together with the extensive PCA overlap (Figure 3), these results provide no evidence of a robust, laterite-shared metabolic response at the present replication. In contrast, the no-soil comparison exceeded chance (244 DEMs), retained three FDR-significant metabolites (Table 4), and achieved Q2 = 0.715. Soil absence, a major departure from the natural pupariation environment, produced a detectable shift, whereas the subtler laterite contrast did not. Two of the three metabolites are level-2 annotations requiring confirmation. The third, ligustrazine (level 1), was ~17-fold lower in no-soil larvae.

Although no laterite metabolite passed FDR control, the direction pattern of the MSI level-1 candidates is biologically plausible and coincides temporally with the phenotype. In the Sanjiang comparison, three central carbon compounds (trans-aconitate, fumarate, and pyruvaldehyde (methylglyoxal)) were all lower (*p* = 0.009–0.019). The Guanlanhu and Chengmai sets were likewise dominated by decreases (15 of 17 and 6 of 8, including ectoine in both). With all q values ≥ 0.45, these are prioritization candidates for targeted assays, not demonstrated changes. Their timing is nonetheless suggestive. The sampling window brackets the prepupal transition in the laterite group but not in the control (19 pprox.. Day 2.4 vs. 19 pprox.. Day 3.4 post-burrowing). Developmental-stage asynchrony is therefore the primary confounder of the metabolomic sampling window: laterite larvae were metamorphosing while control larvae were still wandering, so any day-wise or pooled metabolic difference conflates substrate with developmental stage. Two causal directions are equally compatible with the data and cannot be distinguished here. First, laterite contact may suppress energy and carbon metabolism, contributing to earlier pupariation. Second, laterite-group larvae may have been developmentally ahead within the window so that their metabolomes reflect a more advanced, feeding-arrested prepupal stage rather than a soil effect. Disentangling these explanations requires stage-synchronized sampling and transcriptomic or proteomic evidence, neither of which is available here. The day-resolved patterns are concordant with this interpretation. These patterns comprise 69 cross-laterite shared Day 3 DEMs (67 direction-consistent) and six shared energy-metabolism pathways trending downward. However, they derive from *n* = 2 comparisons without FDR control, and our re-analysis found none significant in laterite day-wise comparisons. They therefore cannot independently support the mechanism.

Finally, the substrate factors are confounded. Acidity, metal content, and physical structure co-vary between laterite and yellow earth and cannot be separated. The recently reported chromosome-level genomes [31,32] make such follow-up feasible, for example, by quantifying ecdysteroid signaling and TCA-cycle enzyme expression along the same aligned timeline.

### 4.4. Comparison with Previous Work

No prior study has used laterite as a substrate. The closest analogues are soil-type-x-moisture experiments on phorid flies and *L. sericata* [15,16]. Tropical studies have linked edaphic context to decomposition and insect succession [17]. Contaminant studies have shown that moisture, malathion, and copper alter blow fly oviposition, development, and immunity [33,34]. Our results fit this picture: substrate chemistry and physics jointly tune blow fly development. They extend it to a soil that dominates large tropical areas yet has been absent from forensic entomology. Monocrotophos exposure altered pupariation timing and disturbed amino acid, carbohydrate, and lipid metabolic gene expression in *C. megacephala* [35], although a direct insecticide challenge is only loosely analogous to a soil matrix. Transcriptomic stress responsiveness [36] and central lipid catabolism in this species [37] provide a plausible backdrop for the candidate directions observed. Methodologically, pairing a coarse-merged comparison (with FDR control) with day-resolved hypothesis generation offers a template for replication-limited exploratory studies.

### 4.5. Forensic Significance: A Potential One-Day Bias Requiring Field Validation

A 24 h pupariation difference matters because developmental-stage-based minimum PMI estimation relies on reference timelines [9,10,11,12]. Timelines generated on one soil type but applied to a different one introduce systematic error. Under the controlled conditions here (29/25 °C, 80% RH), applying yellow earth-derived timelines to a laterite scene could overestimate the minimum PMI by ~1 day for early decomposition windows, because larvae on laterite pupariate ~24 h sooner. This extrapolation is limited to the tested conditions: a single controlled day/night temperature regime (29/25 °C), 80% RH, and one fly population from a single trapping locality in Haikou. Fluctuating temperatures are known to alter developmental rates in this species [12]. Field decomposition proceeds under fluctuating temperatures, variable moisture, and mixed substrates. The effect size, and potentially its direction, may therefore change. Field trials on laterite are required before casework application. The effect is also small compared with temperature, which can shift development by many days. Soil type should thus be regarded as a secondary, region-specific correction. The effect may also propagate into pupal age estimation, where intra-puparial markers are read against timelines [38]. More broadly, multi-omics PMI research shows that molecular clocks can complement developmental ones [39,40,41,42]. The level-1 candidates flagged here are far from validated markers, but they illustrate how soil-informed candidates could be prioritized. Laboratories in laterite-dominated regions should develop region- and soil-specific developmental baselines.

### 4.6. Limitations

This study has several limitations. First, statistical power is limited. Day-wise metabolomic comparisons rested on *n* = 2 per group-day, and even the merged comparisons (*n* = 5–6) are powered only for large effects. Hence, no laterite metabolite survived FDR correction. Post hoc power analysis indicates that detecting a medium effect size (Cohen’s d = 0.5) at 80% power would require *n* ≈ 64 per group. Detecting a large effect (d = 1.0) would require *n* ≈ 17 per group. The current replication is insufficient for definitive metabolomic conclusions. Second, no gut evacuation was performed. Whole-larval metal content, and possibly body weight, includes gut contents, so tissue uptake cannot be separated from gut residues. Third, the substrate factors (acidity, metal content, physical structure) are mutually confounded and cannot be separated without factorial designs. Fourth, most metabolite annotations are MSI level 2 or 3.1 without authentic-standard confirmation. Fifth, all soils came from a single campaign under one set of laboratory conditions without field validation, which limits generalization. Sixth, each treatment group was reared in a single container, so container and treatment are fully confounded and container-level effects (pseudoreplication) cannot be excluded; replication across multiple independent containers per treatment is essential for confirmatory studies. Seventh, soil microbial communities were not characterized. Laterite and yellow earth almost certainly differ in microbial composition, and soil- and gut-associated microbiota can modulate dipteran larval development, nutrition, and metabolite pools; microbial contributions to the observed phenotype, and to candidate metabolites of likely microbial origin such as the osmolyte ectoine, therefore cannot be excluded. Eighth, exchangeable acidity and exchangeable aluminum were not measured, so the biologically active aluminum fraction in these acid soils is unknown.

### 4.7. Future Directions

The priorities are as follows. First, larger replicated experiments (*n* ≥ 12–16 per group) are needed across multiple independent laterite sites, with stage-synchronized sampling and full life-table endpoints (pupariation, survival, eclosion, adult fitness). Second, field trials on laterite should test whether the ~24 h shift persists in casework-relevant settings. Third, factorial designs should separate the acidity, metal-load, and physical-structure components of the laterite effect. Fourth, the level-1 candidates (trans-aconitate, fumarate, pyruvaldehyde, ectoine, medium-chain acylcarnitines) should be quantified by targeted liquid chromatography–tandem mass spectrometry (LC-MS/MS) in adequately powered samples. Fifth, experiments should be replicated across multiple independent rearing containers per treatment to exclude container-level effects. Sixth, soil and larval gut microbiota should be profiled (e.g., 16S rRNA gene amplicon or shotgun metagenomic sequencing) alongside development.

## 5. Conclusions

In this preliminary exploratory study, three Hainan laterites measurably reshaped the development of *C. megacephala*. These soils were strongly acidic (pH 4.17–4.32), Fe/Mn-enriched, and Zn-poor relative to a near-neutral yellow earth. Larvae raised on laterite became significantly heavier (developmental days 4–6) and shorter (days 5–6). They entered the cryptocephalic stage at 130 h vs. 154 h, pupariated 24 h earlier (154 vs. 178 h), and pupariated in shallow (3–5 cm) rather than deep (8–10 cm) soil. Whole-larval Fe and Mn (excluding surface-adhered soil but including gut contents) exceeded controls by ~10.2- and ~4.1-fold. Bioaccumulation factors below 1 indicated no biomagnification, whereas calcium showed BAF > 1 only in the laterite group. The primary merged metabolome analysis (*n* = 5–6 per group; 2975 metabolites; Benjamini–Hochberg correction) found no significant metabolite in any laterite comparison. Unadjusted DEM counts (53–108) fell below the approximate 149 chance expectation. In contrast, the no-soil comparison yielded 244 unadjusted DEMs with three FDR-significant metabolites, indicating a detectable metabolic effect of soil absence itself. The MSI level-1 candidates (e.g., decreased trans-aconitate, fumarate, and pyruvaldehyde in the Sanjiang comparison) are directional hypotheses that coincide with the laterite group’s earlier entry into the cryptocephalic stage. Whether metabolic change drives or merely reflects the developmental advance cannot be resolved here. Under controlled laboratory conditions, the 24 h-earlier pupariation implies a potential ~1-day bias in entomological PMI estimation. This bias must be validated by field decomposition trials before casework application. These baseline data call for larger, stage-synchronized, multi-site studies with targeted metabolite validation.

## Figures and Tables

**Table 1:** Soil pH and metal content of the three laterites and the yellow earth control (mean ± SD, *n* = 4 independent samples per site; *t*-test for each laterite vs. yellow earth; ANOVA among the three laterites).

Parameter	Laterite (Sanjiang)	Laterite (Guanlanhu)	Laterite (Chengmai)	Yellow Earth	*p*, Each Laterite vs. Yellow Earth	*p*, ANOVA Among Laterites
pH	4.23	4.32	4.17	7.45	—	—
Fe (mg/kg)	11,113.8 ± 855.2	11,002.5 ± 812.4	11,205.1 ± 890.7	1930.1 ± 408.5	<0.001 (all three)	0.973
Mn (mg/kg)	79.25 ± 4.58	78.62 ± 4.45	80.11 ± 4.72	21.00 ± 8.93	<0.001 (all three)	0.952
Zn (mg/kg)	9.92 ± 0.98	9.80 ± 1.02	10.05 ± 0.95	21.06 ± 5.16	0.013–0.015	0.961
Al (mg/kg)	142.0 ± 55.8	138.5 ± 52.3	145.2 ± 58.1	98.9 ± 30.7	0.208–0.234	0.986
Ca (mg/kg)	67.9 ± 3.0	68.2 ± 3.3	67.5 ± 3.1	1758.0 ± 1953.0	0.155–0.163	0.993
Mg (mg/kg)	79.1 ± 39.0	80.3 ± 40.5	78.4 ± 38.2	169.0 ± 124.0	0.228–0.241	0.998
K (mg/kg)	2045 ± 941	2008 ± 912	2060 ± 968	2961 ± 878	0.192–0.205	0.997

**Note:** pH was measured in triplicate per sample (averaged values reported; *n* = 4 and the *t*-tests apply to metal rows only).

**Table 2:** Metal content of whole larvae (including gut contents; no gut evacuation) and bioaccumulation factors (BAF) for the three laterites and the yellow earth control (mean ± SD, *n* = 4 per group; *t*-tests of each laterite and of the merged laterite group, *n* = 12, vs. the control; ANOVA among the three laterites).

Element	Laterite (Sanjiang)	Laterite (Guanlanhu)	Laterite (Chengmai)	Yellow Earth	*p*, Each Laterite vs. Control	*p*, Merged Laterite vs. Control	*p*, ANOVA Among Laterites	BAF, Laterite (Merged)	BAF, Yellow Earth
Fe (mg/kg)	489.5 ± 317.2	472.8 ± 305.6	501.2 ± 328.9	48.1 ± 24.8	<0.001 (all three)	<0.001 (t = 8.51)	0.987	0.04	0.03
Mn (mg/kg)	0.212 ± 0.166	0.205 ± 0.158	0.218 ± 0.172	0.052 ± 0.035	0.001 (all three)	<0.001	0.991	0.003	0.003
Zn (mg/kg)	0.153 ± 0.111	0.148 ± 0.105	0.158 ± 0.118	0.140 ± 0.055	0.788–0.895	0.743 (t = 0.33)	0.993	0.02	0.01
Ca (g/kg)	0.18 ± 0.24	0.17 ± 0.22	0.19 ± 0.26	1.09 ± 0.93	0.001 (all three)	<0.001 (t = −4.98)	0.995	2.56	0.73
Mg (g/kg)	1.01 ± 0.75	0.98 ± 0.72	1.04 ± 0.78	0.89 ± 0.39	0.738–0.835	0.654 (t = 0.45)	0.997	12.6	4.9
Al (mg/kg)	<0.01 (below LOD)	<0.01 (below LOD)	<0.01 (below LOD)	<0.01 (below LOD)	—	—	—	—	—

**Note:** BAF = larval tissue concentration/soil concentration (both μg/g, dry weight), computed per sample and averaged; values therefore deviate slightly from ratios of group means (e.g., control Ca). Larvae were rinsed before digestion (excluding surface soil) but not depurated (values include gut contents) and were not dried, so BAF values (dry-weight basis) are approximations. Aluminum was below the limit of detection (LOD, <0.01 mg/kg), with low detection rates and large fluctuation, and was not analyzed further. Potassium was excluded as physiologically implausible (up to 114.8 g/kg; suspected unit or measurement error) and is omitted from the table and all conclusions. t values refer to the merged laterite group (*n* = 12) vs. the control (*n* = 4).

**Table 3:** Primary merged differential analysis (three sampling days pooled per substrate; 2975 metabolites tested per comparison; DEM = differentially expressed metabolite, VIP > 1 and unadjusted *p* < 0.05; FDR = Benjamini–Hochberg-adjusted q < 0.05; PLS-DA = one-component partial least squares–discriminant analysis with leave-one-out cross-validation).

Comparison	*n* (Treat vs. CON = 6)	Unadjusted DEMs (Up/Down)	Random Expectation at α = 0.05	FDR-Significant DEMs	PLS-DA R2Y	PLS-DA Q2 (LOO-CV)
CON vs. Treat1 (Guanlanhu laterite)	6	70 (28/42)	≈149	0	0.845	−0.700
CON vs. Treat2 (Sanjiang laterite)	6	108 (47/61)	≈149	0	0.892	0.037
CON vs. Treat3 (Chengmai laterite)	5	53 (15/38)	≈149	0	0.829	−1.169
CON vs. Treat4 (no soil)	6	244 (94/150)	≈149	3	0.946	0.715

**Table 4:** The three metabolites significant after Benjamini–Hochberg correction (q < 0.05, with VIP > 1) in the merged no-soil comparison (CON vs. Treat4); no metabolite passed this threshold in any laterite comparison.

Metabolite	MSI Level	log2FC (Treat4/CON)	*p* Value	q Value (BH)	VIP	Direction
Isovaleramide	2	+3.26	8.6 × 10^−9^	2.6 × 10^−5^	2.29	Up
6-Amino-1-(2-methoxyethyl)-1,2,3,4-tetrahydropyrimidine-2,4-dione	2	−3.28	1.2 × 10^−6^	0.0018	2.35	Down
Ligustrazine	1	−4.09	1.9 × 10^−5^	0.019	5.13	Down

**Note: 2.** annotations requiring confirmation with authentic standards.

**Table 5:** Candidate Metabolomics Standards Initiative (MSI) level-1 metabolites in the merged laterite comparisons (top entries per comparison by *p* value; complete lists of 17, 15, and 8 candidates in Table S5). All are unadjusted-criterion candidates (VIP > 1 and *p* < 0.05); none is significant after Benjamini–Hochberg correction (q).

Comparison	Metabolite	log2FC (Treat/CON)	*p* Value	q Value (BH)	VIP	Direction
CON vs. Treat1 (Guanlanhu)	4-Hydroxyphenylacetic acid ^‡^	−2.12	0.011	0.98	2.17	Down
	3-Hydroxyphenylacetic acid ^‡^	−2.12	0.011	0.98	2.17	Down
	Ectoine	−1.85	0.013	0.98	2.06	Down
	3-Hydroxyisovaleric acid ^‡^	−0.91	0.014	0.98	1.07	Down
	N-Acetylhistamine	+1.69	0.017	0.98	2.14	Up
	Tryptamine	−2.07	0.026	0.98	2.26	Down
	trans-Aconitic acid	−1.08	0.031	0.98	1.40	Down
	2-Phenyllactic acid ^‡^	−2.52	0.041	0.98	2.75	Down
CON vs. Treat2 (Sanjiang)	4-Cresol sulfate	+6.19	7.6 × 10^−4^	0.45	5.89	Up
	trans-Aconitic acid	−1.41	0.009	0.91	1.49	Down
	3-Methyluridine	+1.18	0.013	0.91	1.13	Up
	N-Acetylglucosamine 6-phosphate	−1.34	0.016	0.91	1.19	Down
	Ligustrazine	−0.94	0.020	0.91	1.18	Down
	N-Acetylcarnosine	−1.99	0.022	0.91	2.40	Down
	N2-Acetyllysine	+1.35	0.023	0.91	1.25	Up
	N5-Acetylornithine ^‡^	−1.55	0.026	0.91	1.42	Down
CON vs. Treat3 (Chengmai)	Ligustrazine	−1.66	0.002	0.99	4.39	Down
	2,4,6-Trihydroxybenzoic acid	−1.11	0.006	0.99	1.22	Down
	N6-Acetyllysine	−0.80	0.007	0.99	1.05	Down
	Ectoine	−2.63	0.011	0.99	3.19	Down
	13(S)-HODE	−1.60	0.014	0.99	1.87	Down
	Phe-Tyr	+0.89	0.017	0.99	1.09	Up
	Kynurenic acid	−1.43	0.037	0.99	1.65	Down
	Crotonoside	+2.55	0.047	0.99	2.61	Up

**Note:** BH = Benjamini–Hochberg; VIP = variable importance in projection; HODE = hydroxyoctadecadienoic acid. ^‡^ Rows with numerically identical statistics within a comparison (further cases in Table S5) are probably multiple annotations of single features. Two further level-1 central carbon metabolites met *p* < 0.05 with VIP ≤ 1 in the Sanjiang comparison: fumaric acid (log2FC = −0.73, *p* = 0.017, q = 0.91, VIP = 0.79) and pyruvaldehyde (log2FC = −0.66, *p* = 0.019, q = 0.91, VIP = 0.71).

## Data Availability

The untargeted metabolomics dataset generated under service project BQ-LYL20260318-NGM-YC (Shanghai BIOTREE Biological Technology Co., Ltd., Shanghai, China) and the data presented in this study are available from the corresponding author on request.

## Supplementary Materials

The following supporting information can be downloaded at: https://www.mdpi.com/article/10.3390/insects17080841/s1, Tables S1–S8 and Figure S1 were supplied as actual files with the submission; Table S1: Summary statistics of soil physicochemical properties (mean ± SD, n = 4 independent samples per site); Table S2: Whole-larval metal content and bioaccumulation factors: group means ± SD for each group (three laterite sites and the yellow-earth control) with ANOVA and t-test results; Table S3: Complete results of the primary merged differential analysis, one table per comparison (S3a, CON vs. Guanlanhu laterite; S3b, CON vs. Sanjiang laterite; S3c, CON vs. Chengmai laterite; S3d, CON vs. no soil): 2975 metabolites each with log2FC, *p* value, Benjamini–Hochberg q value, VIP, and MSI level, with xenobiotic-like level-2/3.1 annotations flagged as probable false positives; Table S4: Day-wise comparisons (n = 2 per group-day): the authors’ recomputed day-wise statistics with Benjamini–Hochberg FDR correction (2975 metabolites across the 11 comparisons); Table S5: Complete MSI level-1 candidate lists of the four merged comparisons (17, 15, 8, and 50 compounds for the Guanlanhu, Sanjiang, Chengmai, and no-soil comparisons, respectively; the two VIP ≤ 1 Sanjiang candidates are given in Table 5 footnote); Table S6: Exploratory KEGG pathway results for the day-wise comparisons (S6a, over-representation analysis: 94 pathway entries with *p* < 0.05 across the 11 comparisons, uncorrected; S6b, GSEA: 1056 gene sets, of which only arachidonic acid metabolism in the CON_3 vs. Treat4_3 comparison was significant after Benjamini–Hochberg correction, adjusted *p* = 0.0258; S6c, MetaboAnalyst topology analysis: 127 pathways, none FDR-significant); Table S7: Phenotype series and endpoints for the three laterite substrates and the yellow earth control (S7a, body weight at seven developmental time points, 22–154 h, mean ± SD, n = 10 per group per time point; S7b, body length at the same seven time points; S7c, phenotype endpoints—cryptocephalic-stage onset, pupariation time, wandering depth—and total protein concentration); Table S8: Reconciliation of the venn-matrix-based and list-based cross-laterite intersections: the vendor matrix (1216-entry union list) omitted three Day 1 DEM names from its universe; the feature-level deduplicated counts recomputed from the per-comparison DEM lists (5, 15, and 69 shared DEMs on Days 1–3; Day 3: 67 of 69 direction-consistent, 40 of 69 not differentially expressed in the no-soil comparison) are used consistently throughout; Figure S1: day-resolved patterns (day-wise DEM counts, cross-laterite shared DEMs per day, and the top shared Day 3 metabolites), reported as hypothesis-generating material.

## Abbreviations

The following abbreviations are used in this manuscript:

**Abbreviation**	**Full name**
ANOVA	Analysis of variance
Arb	Arbitrary units
BAF	Bioaccumulation factor
BCA	Bicinchoninic acid
BH	Benjamini–Hochberg
BLAST	Basic Local Alignment Search Tool
COI	Cytochrome c oxidase subunit I
DA	Differential abundance
DEM	Differentially expressed metabolite
FC	Fold change
FDR	False discovery rate
GSEA	Gene set enrichment analysis
HODE	Hydroxyoctadecadienoic acid
ICP-MS	Inductively coupled plasma mass spectrometry
KEGG	Kyoto Encyclopedia of Genes and Genomes
LC-MS/MS	Liquid chromatography–tandem mass spectrometry
LOO-CV	Leave-one-out cross-validation
LOD	Limit of detection
MSI	Metabolomics Standards Initiative
NES	Normalized enrichment score
OPLS-DA	Orthogonal partial least squares–discriminant analysis
PBS	Phosphate-buffered saline
PCA	Principal component analysis
PLS-DA	Partial least squares–discriminant analysis
PMI	Postmortem interval
QC	Quality control
RH	Relative humidity
RSD	Relative standard deviation
SD	Standard deviation
SNCE	Stepped normalized collision energy
TCA	Tricarboxylic acid (citrate cycle)
TIC	Total ion current
UHPLC-MS/MS	Ultra-high-performance liquid chromatography–tandem mass spectrometry
VIP	Variable importance in projection
