# Supplementary material for: Effects of Tropical Laterite Soils on Larval Development and the Metabolome of the Forensically Important Blow Fly Chrysomya megacephala (Diptera: Calliphoridae): An Exploratory Study
Source: Insects. 2026 Aug 13;17(8):841. doi: 10.3390/insects17080841 (PMC13513139; doi:10.3390/insects17080841)
Supplement: Supplementary file 1 [file insects-17-00841-s001.zip › insects-4487072-supplementary.pdf]

Supplementary Materials

Effects of Tropical Laterite Soils on Larval Development and the Metabolome of the Forensically Important Blow Fly *Chrysomya megacephala* (Diptera: Calliphoridae): A Preliminary Exploratory Study

Insects (MDPI) — Supplementary Tables S1–S8 and Figure S1

Note: Tables S3a–S3d, S4 and S6b exceed practical page limits and are provided in full in the accompanying Excel workbook (Supplementary_Tables.xlsx); their complete captions, column keys and workbook sheet names are given below. All other tables are reproduced in full in this document.

Table S1. Summary statistics of soil physicochemical properties (mean ± SD, n = 4 independent samples per site).

Element	Sanjiang laterite	Guanlanhu laterite	Chengmai laterite	Yellow earth (control)	Laterite-site ANOVA P	Pooled laterite vs control P
pH	4.23	4.32	4.17	7.45	—	—
Fe (mg/kg)	11113.8 ± 855.2	11002.5 ± 812.4	11205.1 ± 890.7	1930.1 ± 408.5	0.973	<0.001
Al (mg/kg)	142.0 ± 55.8	138.5 ± 52.3	145.2 ± 58.1	98.9 ± 30.7	0.986	0.180
Zn (mg/kg)	9.92 ± 0.98	9.80 ± 1.02	10.05 ± 0.95	21.06 ± 5.16	0.961	0.010
Mn (mg/kg)	79.25 ± 4.58	78.62 ± 4.45	80.11 ± 4.72	21.00 ± 8.93	0.952	<0.001
Ca (mg/kg)	67.9 ± 3.0	68.2 ± 3.3	67.5 ± 3.1	1758.0 ± 1953.0	0.993	0.128
Mg (mg/kg)	79.1 ± 39.0	80.3 ± 40.5	78.4 ± 38.2	169.0 ± 124.0	0.998	0.190
K (mg/kg)	2045 ± 941	2008 ± 912	2060 ± 968	2961 ± 878	0.997	0.170

Values are mean ± SD of n = 4 independent soil samples per site. "Laterite-site ANOVA P" = one-way ANOVA across the three laterite sites; "Pooled laterite vs control P" = two-sided Student's t-test of the merged laterite samples against the yellow-earth control. —, not applicable.

Table S2. Whole-larval metal content and bioaccumulation factors (BAF): group means ± SD for each group (three laterite sites and the yellow-earth control) with ANOVA and t-test results.

Element	Sanjiang laterite	Guanlanhu laterite	Chengmai laterite	Yellow earth (control)	Laterite-site ANOVA P	Pooled laterite vs control P	BAF laterite (merged)	BAF control
Fe (mg/kg)	489.5 ± 317.2	472.8 ± 305.6	501.2 ± 328.9	48.1 ± 24.8	0.987 (ns)	<0.001 (t=8.51)	0.04	0.03
Mn (mg/kg)	0.212 ± 0.166	0.205 ± 0.158	0.218 ± 0.172	0.052 ± 0.035	0.991 (ns)	<0.001	0.003	0.003
Zn (mg/kg)	0.153 ± 0.111	0.148 ± 0.105	0.158 ± 0.118	0.140 ± 0.055	0.993 (ns)	0.743	0.02	0.01
Ca (g/kg)	0.18 ± 0.24	0.17 ± 0.22	0.19 ± 0.26	1.09 ± 0.93	0.995 (ns)	<0.001 (t=-4.98)	2.56	0.73
Mg (g/kg)	1.01 ± 0.75	0.98 ± 0.72	1.04 ± 0.78	0.89 ± 0.39	0.997 (ns)	0.654	12.6	4.9
Al	below LOD (<0.01 mg/kg)	below LOD	below LOD	below LOD	—	—	—	—
K	excluded: implausible value (114.8 g/kg), unit/measurement error suspected	—	—	—	—	—	—	—

Values are mean ± SD (n = 4 independent larval samples per group). BAF = whole-larval concentration / soil concentration. Al was below the limit of detection (LOD < 0.01 mg/kg) in all groups. The larval K value was excluded because the recorded value (114.8 g/kg) is physiologically implausible and a unit or measurement error is suspected. ns, not significant; —, not applicable/not computed.

Tables S3a–S3d. Complete results of the primary merged differential analysis (one table per comparison): 2,975 metabolites each with log2FC, p value, Benjamini–Hochberg q value, VIP, and MSI level, with xenobiotic-like level-2/3.1 annotations flagged as probable false positives.

Note： Given that the table exceeds 2,000 rows, it is not presented here in full to avoid distracting from the core findings. Readers may request the complete table from the corresponding author.

Table S3a. Primary merged differential analysis, CON vs. Guanlanhu laterite (Treat1) (CON n = 6, Treat1 n = 6). The complete table (2,975 metabolites) is provided in the accompanying Excel workbook Supplementary_Tables.xlsx, sheet "S3a Pooled CONvsGuanlanhu".

Table S3b. Primary merged differential analysis, CON vs. Sanjiang laterite (Treat2) (CON n = 6, Treat2 n = 6). The complete table (2,975 metabolites) is provided in the accompanying Excel workbook Supplementary_Tables.xlsx, sheet "S3b Pooled CONvsSanjiang".

Table S3c. Primary merged differential analysis, CON vs. Chengmai laterite (Treat3) (CON n = 6, Treat3 n = 5). The complete table (2,975 metabolites) is provided in the accompanying Excel workbook Supplementary_Tables.xlsx, sheet "S3c Pooled CONvsChengmai".

Table S3d. Primary merged differential analysis, CON vs. no soil (Treat4) (CON n = 6, Treat4 n = 6). The complete table (2,975 metabolites) is provided in the accompanying Excel workbook Supplementary_Tables.xlsx, sheet "S3d Pooled CONvsNoSoil".

Column key for Tables S3a–S3d — ID: feature index; compound: metabolite annotation; level: MSI confidence level (1/2/3.1); mean_CON / mean_Treat: group mean peak areas; log2FC: log2 fold change (treatment vs yellow-earth control); t, p: Student's t-test; q_BH: Benjamini–Hochberg false discovery rate; VIP: variable importance in projection from a one-component PLS-DA; direction: regulation vs control; DEM_VIP1_p005: differentially expressed metabolite at VIP > 1 and p < 0.05 (unadjusted); DEM_FDR005: significant after Benjamini–Hochberg correction (q_BH < 0.05); xenobiotic_like_flag: pesticide/drug-like level-2/3.1 annotation treated as a probable annotation false positive (keyword-based heuristic matching a curated list of agrochemical and pharmaceutical name fragments; 40 annotations flagged per table).

Table S4. Day-wise comparisons (n = 2 per group-day): the authors' recomputed day-wise statistics with Benjamini–Hochberg FDR correction (2,975 metabolites across the 11 comparisons; 32,725 rows). The complete table is provided in Supplementary_Tables.xlsx, sheet "Table S4 Per-day with FDR".

Note： Given that the table exceeds 30,000 rows, it is not presented here in full to avoid distracting from the core findings. Readers may request the complete table from the corresponding author.

Day-wise groups contained n = 2 larvae per group per day (Chengmai Day 3: n = 1; no CON_3 vs Treat3_3 model was computed for the single-replicate cell). Columns: day (1–3 post-burrowing), comparison, ID, compound, level (MSI), log2FC, p, q_BH. No metabolite was significant after FDR correction in any day-wise comparison except a single metabolite in the Day-3 no-soil comparison; all day-wise results are hypothesis-generating.

Table S5. Complete MSI level-1 candidate lists of the four merged comparisons (17, 15, 8, and 50 compounds for the Guanlanhu, Sanjiang, Chengmai, and no-soil comparisons, respectively).

Compound	Comparison	log2FC	p	q_BH	VIP	Direction
4-Hydroxyphenylacetic acid	Guanlanhu	-2.12	0.0112	0.9797	2.17	down
3-Hydroxyphenylacetic acid	Guanlanhu	-2.12	0.0112	0.9797	2.17	down
Ectoine	Guanlanhu	-1.85	0.0134	0.9797	2.06	down
3-Hydroxyisovaleric acid	Guanlanhu	-0.91	0.0143	0.9797	1.07	down
3-Hydroxyvaleric acid	Guanlanhu	-0.91	0.0143	0.9797	1.07	down
N-Acetylhistamine	Guanlanhu	1.69	0.0173	0.9797	2.14	up
Tryptamine	Guanlanhu	-2.07	0.026	0.9797	2.26	down
trans-Aconitic acid	Guanlanhu	-1.08	0.0313	0.9797	1.40	down
1-Aminopropan-2-ol	Guanlanhu	-1.23	0.0332	0.9797	1.65	down
PG(18:1/18:1(9Z))	Guanlanhu	-0.91	0.0348	0.9797	1.03	down
2-Phenyllactic acid	Guanlanhu	-2.52	0.0414	0.9797	2.75	down
4-Ethoxybenzoic acid	Guanlanhu	-2.52	0.0414	0.9797	2.75	down
Ethyl 4-hydroxybenzoate	Guanlanhu	-2.52	0.0414	0.9797	2.75	down
Ethyl 3-hydroxybenzoate	Guanlanhu	-2.52	0.0414	0.9797	2.75	down
cis-8,11,14-Eicosatrienoic acid	Guanlanhu	-1.19	0.042	0.9797	1.22	down
5Z,8Z,11Z-Eicosatrienoic acid	Guanlanhu	-1.19	0.042	0.9797	1.22	down
2-Aminoheptanoic acid	Guanlanhu	2.37	0.0458	0.9797	2.50	up
4-Cresol sulfate	Sanjiang	6.19	0.0008	0.4495	5.89	up
trans-Aconitic acid	Sanjiang	-1.41	0.0088	0.9118	1.49	down
3-Methyluridine	Sanjiang	1.18	0.0126	0.9118	1.13	up
N-Acetylglucosamine 6-Phosphate	Sanjiang	-1.34	0.0163	0.9118	1.19	down
Ligustrazine	Sanjiang	-0.94	0.0197	0.9118	1.18	down
N-Acetylcarnosine	Sanjiang	-1.99	0.0224	0.9118	2.40	down
N2-Acetyllysine	Sanjiang	1.35	0.0233	0.9118	1.25	up
N5-acetyl-l-ornithine	Sanjiang	-1.55	0.0264	0.9118	1.42	down
Theanine	Sanjiang	-1.55	0.0264	0.9118	1.42	down
Gly-Val	Sanjiang	-1.55	0.0264	0.9118	1.42	down

N2-Acetylornithine	Sanjiang	-1.55	0.0264	0.9118	1.42	down
SM(d18:1/17:0)	Sanjiang	-1.12	0.0273	0.9118	1.08	down
Octanoylcarnitine (Car(8:0))	Sanjiang	2.08	0.0344	0.9198	1.74	up
AG 1295	Sanjiang	-1.24	0.0351	0.9198	1.24	down
Hexanoylcarnitine (Car(6:0))	Sanjiang	2.05	0.0452	0.9198	1.78	up
Ligustrazine	Chengmai	-1.66	0.0021	0.989	4.39	down
2,4,6-Trihydroxybenzoic acid	Chengmai	-1.11	0.006	0.989	1.22	down
N6-Acetyllysine	Chengmai	-0.80	0.0067	0.989	1.05	down
Ectoine	Chengmai	-2.63	0.0107	0.989	3.19	down
13(S)-HODE	Chengmai	-1.60	0.0142	0.989	1.87	down
Phe-Tyr	Chengmai	0.89	0.0167	0.989	1.09	up
Kynurenic acid	Chengmai	-1.43	0.0375	0.989	1.65	down
Crotonoside	Chengmai	2.55	0.0467	0.989	2.61	up
Ligustrazine	No soil	-4.09	1.89e-05	0.0187	5.13	down
Creatine	No soil	1.68	0.0005	0.0748	1.19	up
Imidazol-1-yl-acetic acid	No soil	2.71	0.0015	0.13	2.30	up
4-Aminobutyric acid (GABA)	No soil	1.53	0.0023	0.1313	1.06	up
Methyl β-D-glucopyranoside	No soil	-1.35	0.0029	0.1313	1.11	down
Methyl β-D-Galactopyranoside	No soil	-1.35	0.0029	0.1313	1.11	down
Indole-3-carboxaldehyde	No soil	-1.68	0.0034	0.1313	1.28	down
N5-acetyl-l-ornithine	No soil	1.50	0.0035	0.1313	1.11	up
Gly-Val	No soil	1.50	0.0035	0.1313	1.11	up
N2-Acetylornithine	No soil	1.50	0.0035	0.1313	1.11	up
Theanine	No soil	1.50	0.0035	0.1313	1.11	up
Sarcosine	No soil	1.58	0.0052	0.1521	1.02	up
Alanine	No soil	1.58	0.0052	0.1521	1.02	up
N-Acetylcarnosine	No soil	2.05	0.0066	0.1736	1.31	up
5-Hydroxytryptophan	No soil	2.30	0.0066	0.1736	1.73	up
Sorbose	No soil	2.00	0.0069	0.1764	1.45	up
Cytidine 5'-monophosphate (CMP)	No soil	-1.64	0.008	0.1865	1.12	down
Uridine diphosphate glucose (UDP-glucose)	No soil	-1.27	0.0082	0.1865	1.47	down
12-Hydroxystearic acid	No soil	2.18	0.0089	0.1916	1.64	up
Quinic acid	No soil	-2.20	0.0093	0.1926	1.51	down
2-Hydroxystearic acid	No soil	2.26	0.0095	0.1926	1.64	up
3-Ureidopropionic acid	No soil	-2.18	0.0097	0.1926	1.43	down
Diglycine	No soil	-2.18	0.0097	0.1926	1.43	down
Phenylacetylglycine	No soil	-2.41	0.0109	0.2019	1.59	down
2-Methylhippuric acid	No soil	-2.41	0.0109	0.2019	1.59	down
Fagomine	No soil	-2.01	0.012	0.2077	1.28	down
Creatinine	No soil	2.15	0.0123	0.2097	1.78	up
1-Methylinosine	No soil	1.01	0.0151	0.235	1.08	up
Phenylacetic acid	No soil	-1.59	0.0162	0.2396	1.45	down
2-Methylbenzoic acid	No soil	-1.59	0.0162	0.2396	1.45	down
4-Methylbenzoic-acid	No soil	-1.59	0.0162	0.2396	1.45	down
Capric acid	No soil	1.55	0.0162	0.2396	1.09	up
beta-Guanidinopropionic acid	No soil	2.95	0.0185	0.2492	1.97	up
3,5-Dimethoxybenzoic acid	No soil	-1.48	0.0191	0.2519	1.85	down
Veratric acid	No soil	-1.48	0.0191	0.2519	1.85	down
N1,N8-Diacetylspermidine	No soil	1.63	0.0205	0.2565	1.31	up
Ornithine	No soil	1.83	0.022	0.268	1.13	up
NAE(18:1)	No soil	1.87	0.0231	0.2765	1.38	up
Hexadecanedioic acid	No soil	1.75	0.0266	0.2964	1.05	up
4-Hydroxyphenylacetic acid	No soil	-1.69	0.0301	0.3101	1.49	down
3-Hydroxyphenylacetic acid	No soil	-1.69	0.0301	0.3101	1.49	down
Uridine 5'-monophosphate (UMP)	No soil	-1.77	0.0325	0.3183	1.22	down
Hypoxanthine	No soil	1.64	0.035	0.3247	1.16	up
2-Methylbutyrylglycine	No soil	-1.38	0.0375	0.3253	1.08	down
Isovalerylglycine	No soil	-1.38	0.0375	0.3253	1.08	down
2-Aminoisobutyric acid	No soil	-1.73	0.0375	0.3253	1.02	down
2-Aminobutyric acid	No soil	-1.73	0.0375	0.3253	1.02	down
Homocitric acid	No soil	-1.55	0.0375	0.3253	1.03	down
Hydroxyphenylacetylglycine	No soil	-2.80	0.0472	0.3572	1.70	down

Allopurinol	No soil	-1.34	0.048	0.3588	1.22	down

Candidates are MSI level-1 metabolites with VIP > 1 and p < 0.05 in the merged comparison (unadjusted); none remained significant after Benjamini–Hochberg correction. The two Sanjiang candidates with VIP ≤ 1 discussed in the main text are given in the footnote of main-text Table 5.

Table S6a. Exploratory KEGG over-representation analysis of the day-wise comparisons: 94 pathway entries with p < 0.05 across the 11 comparisons (uncorrected; hypothesis-generating).

Comparison	KEGG ID	Pathway	Rich factor	p	Up	Down	DA score
CON_1vsTreat1_1	dme00360	Phenylalanine metabolism	0.070	2.88e-05	1	3	-0.167
CON_1vsTreat1_1	dme00290	Valine, leucine and isoleucine biosynthesis	0.120	6.37e-05	3	0	0.273
CON_1vsTreat1_1	dme01210	2-Oxocarboxylic acid metabolism	0.028	0.0002	5	0	0.179
CON_1vsTreat1_1	dme00240	Pyrimidine metabolism	0.038	0.0003	2	2	0.000
CON_1vsTreat1_1	dme01230	Biosynthesis of amino acids	0.026	0.0003	5	0	0.109
CON_1vsTreat1_1	dme00470	D-Amino acid metabolism	0.042	0.0015	3	0	0.158
CON_1vsTreat1_1	dme01040	Biosynthesis of unsaturated fatty acids	0.030	0.0038	1	2	-0.100
CON_1vsTreat1_1	dme00330	Arginine and proline metabolism	0.024	0.007	3	0	0.136
CON_1vsTreat1_1	dme00410	beta-Alanine metabolism	0.035	0.0141	1	1	0.000
CON_1vsTreat1_1	dme00640	Propanoate metabolism	0.029	0.0203	1	1	0.000
CON_1vsTreat1_1	dme00350	Tyrosine metabolism	0.020	0.0403	0	2	-0.111
CON_1vsTreat2_1	dme00340	Histidine metabolism	0.073	2.84e-06	2	2	0.000
CON_1vsTreat2_1	dme00232	Caffeine metabolism	0.107	1.83e-05	3	0	0.500
CON_1vsTreat2_1	dme00360	Phenylalanine metabolism	0.035	0.0052	0	2	-0.167
CON_1vsTreat2_1	dme00470	D-Amino acid metabolism	0.028	0.0082	1	1	0.000
CON_1vsTreat2_1	dme00350	Tyrosine metabolism	0.020	0.0153	0	2	-0.111
CON_1vsTreat2_1	dme00270	Cysteine and methionine metabolism	0.019	0.0168	1	1	0.000
CON_1vsTreat3_1	dme00640	Propanoate metabolism	0.029	0.0021	0	2	-0.167
CON_1vsTreat3_1	dme01210	2-Oxocarboxylic acid metabolism	0.011	0.0135	1	1	0.000
CON_1vsTreat3_1	dme01230	Biosynthesis of amino acids	0.010	0.0159	1	1	0.000
CON_1vsTreat3_1	dme00290	Valine, leucine and isoleucine biosynthesis	0.040	0.0253	0	1	-0.091
CON_1vsTreat3_1	dme00232	Caffeine metabolism	0.036	0.0284	0	1	-0.167
CON_1vsTreat4_1	dme00260	Glycine, serine and threonine metabolism	0.040	0.0004	2	1	0.048
CON_1vsTreat4_1	dme00590	Arachidonic acid metabolism	0.032	0.0008	2	1	0.167
CON_1vsTreat4_1	dme01210	2-Oxocarboxylic acid metabolism	0.017	0.0054	1	2	-0.036
CON_1vsTreat4_1	dme01230	Biosynthesis of amino acids	0.015	0.0069	1	2	-0.022
CON_1vsTreat4_1	dme00470	D-Amino acid metabolism	0.028	0.0093	1	1	0.000
CON_1vsTreat4_1	dme00564	Glycerophospholipid metabolism	0.017	0.0244	0	2	-0.133
CON_2vsTreat1_2	dme00400	Phenylalanine, tyrosine and tryptophan biosynthesis	0.026	0.0296	0	1	-0.143
CON_2vsTreat1_2	dme00600	Sphingolipid metabolism	0.015	0.0497	1	0	0.100
CON_2vsTreat2_2	dme00310	Lysine degradation	0.033	0.0029	2	1	0.067
CON_2vsTreat2_2	dme01232	Nucleotide metabolism	0.025	0.0061	2	1	0.034
CON_2vsTreat2_2	dme00340	Histidine metabolism	0.036	0.0132	2	0	0.125
CON_2vsTreat2_2	dme00600	Sphingolipid metabolism	0.030	0.0186	2	0	0.200
CON_2vsTreat2_2	dme00230	Purine metabolism	0.015	0.0233	2	1	0.036
CON_2vsTreat2_2	dme00240	Pyrimidine metabolism	0.019	0.0432	1	1	0.000
CON_2vsTreat3_2	dme00310	Lysine degradation	0.022	0.0112	1	1	0.000
CON_2vsTreat3_2	dme00520	Amino sugar and nucleotide sugar metabolism	0.018	0.0169	0	2	-0.182
CON_2vsTreat3_2	dme01230	Biosynthesis of amino acids	0.010	0.0468	1	1	0.000
CON_2vsTreat4_2	dme01230	Biosynthesis of amino acids	0.036	0.0002	5	2	0.065
CON_2vsTreat4_2	dme01040	Biosynthesis of unsaturated fatty acids	0.050	0.0004	5	0	0.500
CON_2vsTreat4_2	dme00350	Tyrosine metabolism	0.050	0.0004	1	4	-0.167
CON_2vsTreat4_2	dme00500	Starch and sucrose metabolism	0.058	0.0009	0	4	-0.571
CON_2vsTreat4_2	dme00470	D-Amino acid metabolism	0.056	0.0011	4	0	0.211
CON_2vsTreat4_2	dme00220	Arginine biosynthesis	0.070	0.0025	3	0	0.250
CON_2vsTreat4_2	dme00330	Arginine and proline metabolism	0.032	0.008	3	1	0.091
CON_2vsTreat4_2	dme00061	Fatty acid biosynthesis	0.042	0.0104	2	1	0.125
CON_2vsTreat4_2	dme00260	Glycine, serine and threonine metabolism	0.040	0.0121	2	1	0.048
CON_2vsTreat4_2	dme00430	Taurine and hypotaurine metabolism	0.061	0.0187	1	1	0.000
CON_2vsTreat4_2	dme00670	One carbon pool by folate	0.039	0.042	2	0	0.182
CON_2vsTreat4_2	dme00340	Histidine metabolism	0.036	0.0481	2	0	0.125
CON_3vsTreat1_3	dme00620	Pyruvate metabolism	0.049	0.0002	0	4	-0.500
CON_3vsTreat1_3	dme00250	Alanine, aspartate and glutamate metabolism	0.053	0.001	2	1	0.062
CON_3vsTreat1_3	dme00360	Phenylalanine metabolism	0.053	0.001	0	3	-0.250
CON_3vsTreat1_3	dme00350	Tyrosine metabolism	0.030	0.0048	0	3	-0.167
CON_3vsTreat1_3	dme00430	Taurine and hypotaurine metabolism	0.061	0.0057	1	1	0.000

CON_3vsTreat1_3	dme00220	Arginine biosynthesis	0.047	0.0095	1	1	0.000
CON_3vsTreat1_3	dme00340	Histidine metabolism	0.036	0.0153	2	0	0.125
CON_3vsTreat1_3	dme00650	Butanoate metabolism	0.033	0.0186	1	1	0.000
CON_3vsTreat1_3	dme00020	Citrate cycle (TCA cycle)	0.032	0.0198	0	2	-0.286
CON_3vsTreat1_3	dme01240	Biosynthesis of cofactors	0.011	0.021	1	4	-0.046
CON_3vsTreat1_3	dme00640	Propanoate metabolism	0.029	0.0234	0	2	-0.167
CON_3vsTreat1_3	dme01210	2-Oxocarboxylic acid metabolism	0.017	0.0235	1	2	-0.036
CON_3vsTreat1_3	dme00760	Nicotinate and nicotinamide metabolism	0.027	0.0267	0	2	-0.125
CON_3vsTreat1_3	dme00051	Fructose and mannose metabolism	0.024	0.0338	0	2	-0.250
CON_3vsTreat1_3	dme00630	Glyoxylate and dicarboxylate metabolism	0.021	0.0439	1	1	0.000
CON_3vsTreat1_3	dme01200	Carbon metabolism	0.013	0.0468	1	2	-0.040
CON_3vsTreat2_3	dme00260	Glycine, serine and threonine metabolism	0.053	7.22e-05	0	4	-0.190
CON_3vsTreat2_3	dme00620	Pyruvate metabolism	0.037	0.0019	0	3	-0.375
CON_3vsTreat2_3	dme01240	Biosynthesis of cofactors	0.011	0.0128	0	5	-0.077
CON_3vsTreat2_3	dme00020	Citrate cycle (TCA cycle)	0.032	0.0158	0	2	-0.286
CON_3vsTreat2_3	dme00760	Nicotinate and nicotinamide metabolism	0.027	0.0214	0	2	-0.125
CON_3vsTreat2_3	dme01230	Biosynthesis of amino acids	0.015	0.0214	0	3	-0.065
CON_3vsTreat2_3	dme01200	Carbon metabolism	0.013	0.0346	0	3	-0.120
CON_3vsTreat2_3	dme00630	Glyoxylate and dicarboxylate metabolism	0.021	0.0353	0	2	-0.118
CON_3vsTreat2_3	dme00240	Pyrimidine metabolism	0.019	0.0401	0	2	-0.100
CON_3vsTreat4_3	dme01210	2-Oxocarboxylic acid metabolism	0.044	4.13e-06	0	8	-0.286
CON_3vsTreat4_3	dme01230	Biosynthesis of amino acids	0.041	7.77e-06	0	8	-0.174
CON_3vsTreat4_3	dme00330	Arginine and proline metabolism	0.048	4.68e-05	2	4	-0.091
CON_3vsTreat4_3	dme00290	Valine, leucine and isoleucine biosynthesis	0.120	0.0003	0	3	-0.273
CON_3vsTreat4_3	dme00470	D-Amino acid metabolism	0.056	0.0006	0	4	-0.211
CON_3vsTreat4_3	dme00430	Taurine and hypotaurine metabolism	0.091	0.0007	0	3	-0.375
CON_3vsTreat4_3	dme01240	Biosynthesis of cofactors	0.019	0.0007	2	7	-0.077
CON_3vsTreat4_3	dme00220	Arginine biosynthesis	0.070	0.0015	0	3	-0.250
CON_3vsTreat4_3	dme00340	Histidine metabolism	0.055	0.0031	0	3	-0.188
CON_3vsTreat4_3	dme00280	Valine, leucine and isoleucine degradation	0.041	0.0072	0	3	-0.375
CON_3vsTreat4_3	dme00740	Riboflavin metabolism	0.062	0.0127	1	1	0.000
CON_3vsTreat4_3	dme00350	Tyrosine metabolism	0.030	0.0163	1	2	-0.056
CON_3vsTreat4_3	dme00770	Pantothenate and CoA biosynthesis	0.045	0.0232	0	2	-0.182
CON_3vsTreat4_3	dme00480	Glutathione metabolism	0.025	0.0258	1	2	-0.125
CON_3vsTreat4_3	dme00250	Alanine, aspartate and glutamate metabolism	0.035	0.0374	0	2	-0.125
CON_3vsTreat4_3	dme00360	Phenylalanine metabolism	0.035	0.0374	0	2	-0.167
CON_3vsTreat4_3	dme00410	beta-Alanine metabolism	0.035	0.0374	1	1	0.000
CON_3vsTreat4_3	dme00650	Butanoate metabolism	0.033	0.0423	0	2	-0.200

Pathways were mapped to the Drosophila melanogaster KEGG library (dme). p values are unadjusted (over-representation analysis of the vendor day-wise DEM sets, n = 2 per group-day); no entry was subjected to multiple-testing correction; results are hypothesis-generating.

Table S6b. Exploratory KEGG gene-set enrichment analysis (GSEA) of the day-wise comparisons: 1,056 gene sets. The complete table is provided in Supplementary_Tables.xlsx, sheet “S6b GSEA”.

Note： Given that the table exceeds the limits, it is not presented here in full to avoid distracting from the core findings. Readers may request the complete table from the corresponding author.

Columns: comparison, ID (KEGG pathway), Description, setSize, enrichmentScore, NES, pvalue, p.adjust (Benjamini–Hochberg). Only arachidonic acid metabolism in the CON_3 vs Treat4_3 comparison remained significant after Benjamini–Hochberg correction (adjusted p = 0.0258).

Table S6c. Exploratory MetaboAnalyst pathway-topology analysis of the day-wise comparisons: 127 pathways; none FDR-significant.

Comparison	Pathway	Total	Hits	Raw p	Holm adjust	FDR	Impact
CON_1vsTreat1_1	Phenylalanine metabolism	10	2	0.0289	1	1	0.000
CON_1vsTreat1_1	Biotin metabolism	5	1	0.131	1	1	0.000
CON_1vsTreat1_1	Nicotinate and nicotinamide metabolism	9	1	0.2238	1	1	0.185
CON_1vsTreat1_1	Arachidonic acid metabolism	10	1	0.2454	1	1	0.000
CON_1vsTreat1_1	Arginine and proline metabolism	37	2	0.2726	1	1	0.155
CON_1vsTreat1_1	Pantothenate and CoA biosynthesis	12	1	0.287	1	1	0.000
CON_1vsTreat1_1	beta-Alanine metabolism	13	1	0.3069	1	1	0.000
CON_1vsTreat1_1	Lysine degradation	17	1	0.3814	1	1	0.000
CON_1vsTreat1_1	Glutathione metabolism	26	1	0.522	1	1	0.000

CON_1vsTreat1_1	Tyrosine metabolism	30	1	0.574	1	1	0.000
CON_1vsTreat1_1	Valine, leucine and isoleucine degradation	35	1	0.6314	1	1	0.000
CON_1vsTreat1_1	Pyrimidine metabolism	41	1	0.6905	1	1	0.072
CON_1vsTreat1_1	Aminoacyl-tRNA biosynthesis	67	1	0.8567	1	1	0.000
CON_1vsTreat2_1	Histidine metabolism	7	1	0.0867	1	1	0.000
CON_1vsTreat2_1	Caffeine metabolism	10	1	0.1217	1	1	0.000
CON_1vsTreat2_1	beta-Alanine metabolism	13	1	0.1554	1	1	0.000
CON_1vsTreat2_1	Tryptophan metabolism	23	1	0.2595	1	1	0.000
CON_1vsTreat2_1	Alanine, aspartate and glutamate metabolism	23	1	0.2595	1	1	0.152
CON_1vsTreat2_1	Cysteine and methionine metabolism	25	1	0.2788	1	1	0.122
CON_1vsTreat2_1	Tyrosine metabolism	30	1	0.3251	1	1	0.000
CON_1vsTreat2_1	Arginine and proline metabolism	37	1	0.3853	1	1	0.000
CON_1vsTreat2_1	Aminoacyl-tRNA biosynthesis	67	1	0.5914	1	1	0.000
CON_1vsTreat3_1	Glyoxylate and dicarboxylate metabolism	16	1	0.134	1	1	0.000
CON_1vsTreat3_1	Valine, leucine and isoleucine degradation	35	1	0.2722	1	1	0.000
CON_1vsTreat3_1	Purine metabolism	64	1	0.4456	1	1	0.014
CON_1vsTreat4_1	Glyoxylate and dicarboxylate metabolism	16	1	0.1478	1	1	0.000
CON_2vsTreat1_2	Amino sugar and nucleotide sugar metabolism	34	1	0.2655	1	1	0.003
CON_2vsTreat2_2	Sphingolipid metabolism	18	2	0.0858	1	1	0.095
CON_2vsTreat2_2	Biotin metabolism	5	1	0.131	1	1	0.000
CON_2vsTreat2_2	One carbon pool by folate	8	1	0.2015	1	1	0.000
CON_2vsTreat2_2	Purine metabolism	64	3	0.2578	1	1	0.016
CON_2vsTreat2_2	Folate biosynthesis	16	1	0.3636	1	1	0.000
CON_2vsTreat2_2	Glyoxylate and dicarboxylate metabolism	16	1	0.3636	1	1	0.083
CON_2vsTreat2_2	Lysine degradation	17	1	0.3814	1	1	0.000
CON_2vsTreat2_2	Starch and sucrose metabolism	17	1	0.3814	1	1	0.003
CON_2vsTreat2_2	Amino sugar and nucleotide sugar metabolism	34	1	0.6206	1	1	0.000
CON_2vsTreat2_2	Arginine and proline metabolism	37	1	0.6522	1	1	0.000
CON_2vsTreat2_2	Pyrimidine metabolism	41	1	0.6905	1	1	0.029
CON_2vsTreat2_2	Aminoacyl-tRNA biosynthesis	67	1	0.8567	1	1	0.000
CON_2vsTreat3_2	Biotin metabolism	5	1	0.058	1	1	0.000
CON_2vsTreat3_2	Amino sugar and nucleotide sugar metabolism	34	2	0.0585	1	1	0.030
CON_2vsTreat3_2	Cyanoamino acid metabolism	6	1	0.0692	1	1	0.000
CON_2vsTreat3_2	beta-Alanine metabolism	13	1	0.1443	1	1	0.000
CON_2vsTreat3_2	Folate biosynthesis	16	1	0.1748	1	1	0.000
CON_2vsTreat3_2	Lysine degradation	17	1	0.1847	1	1	0.000
CON_2vsTreat3_2	Propanoate metabolism	18	1	0.1945	1	1	0.167
CON_2vsTreat3_2	Tryptophan metabolism	23	1	0.242	1	1	0.000
CON_2vsTreat3_2	Glutathione metabolism	26	1	0.2693	1	1	0.015
CON_2vsTreat3_2	Arginine and proline metabolism	37	1	0.3617	1	1	0.046
CON_2vsTreat3_2	Purine metabolism	64	1	0.5451	1	1	0.010
CON_2vsTreat3_2	Aminoacyl-tRNA biosynthesis	67	1	0.5621	1	1	0.000
CON_2vsTreat4_2	Starch and sucrose metabolism	17	3	0.0547	1	1	0.120
CON_2vsTreat4_2	Arginine and proline metabolism	37	4	0.1227	1	1	0.105
CON_2vsTreat4_2	Ubiquinone and other terpenoid-quinone biosynthesis	3	1	0.149	1	1	0.000
CON_2vsTreat4_2	Folate biosynthesis	16	2	0.2023	1	1	0.000
CON_2vsTreat4_2	Tyrosine metabolism	30	3	0.2038	1	1	0.188
CON_2vsTreat4_2	Biotin metabolism	5	1	0.236	1	1	0.000
CON_2vsTreat4_2	Cyanoamino acid metabolism	6	1	0.2762	1	1	0.000
CON_2vsTreat4_2	alpha-Linolenic acid metabolism	8	1	0.3504	1	1	0.000
CON_2vsTreat4_2	One carbon pool by folate	8	1	0.3504	1	1	0.000
CON_2vsTreat4_2	Phenylalanine metabolism	10	1	0.4172	1	1	0.000
CON_2vsTreat4_2	beta-Alanine metabolism	13	1	0.5048	1	1	0.000
CON_2vsTreat4_2	Pentose and glucuronate interconversions	14	1	0.5311	1	1	0.000
CON_2vsTreat4_2	Lysine degradation	17	1	0.6019	1	1	0.000
CON_2vsTreat4_2	Propanoate metabolism	18	1	0.6231	1	1	0.167
CON_2vsTreat4_2	Alanine, aspartate and glutamate metabolism	23	1	0.7135	1	1	0.000
CON_2vsTreat4_2	Tryptophan metabolism	23	1	0.7135	1	1	0.000
CON_2vsTreat4_2	Glycine, serine and threonine metabolism	25	1	0.7434	1	1	0.000
CON_2vsTreat4_2	Glutathione metabolism	26	1	0.7571	1	1	0.015
CON_2vsTreat4_2	Aminoacyl-tRNA biosynthesis	67	2	0.8803	1	1	0.000
CON_2vsTreat4_2	Pyrimidine metabolism	41	1	0.8945	1	1	0.000
CON_2vsTreat4_2	Purine metabolism	64	1	0.9714	1	1	0.010
CON_3vsTreat1_3	Alanine, aspartate and glutamate metabolism	23	3	0.021	1	1	0.280
CON_3vsTreat1_3	D-Glutamine and D-glutamate metabolism	5	1	0.1266	1	1	1.000

CON_3vsTreat1_3	Histidine metabolism	7	1	0.1728	1	1	0.000
CON_3vsTreat1_3	Nitrogen metabolism	7	1	0.1728	1	1	0.000
CON_3vsTreat1_3	Tyrosine metabolism	30	2	0.1888	1	1	0.000
CON_3vsTreat1_3	Amino sugar and nucleotide sugar metabolism	34	2	0.2285	1	1	0.098
CON_3vsTreat1_3	Arginine and proline metabolism	37	2	0.2587	1	1	0.120
CON_3vsTreat1_3	Pantothenate and CoA biosynthesis	12	1	0.2782	1	1	0.000
CON_3vsTreat1_3	Valine, leucine and isoleucine biosynthesis	13	1	0.2977	1	1	0.333
CON_3vsTreat1_3	Sphingolipid metabolism	18	1	0.3876	1	1	0.012
CON_3vsTreat1_3	Fructose and mannose metabolism	18	1	0.3876	1	1	0.085
CON_3vsTreat1_3	Citrate cycle (TCA cycle)	20	1	0.4204	1	1	0.027
CON_3vsTreat1_3	Butanoate metabolism	21	1	0.4362	1	1	0.000
CON_3vsTreat1_3	Porphyrin and chlorophyll metabolism	23	1	0.4665	1	1	0.000
CON_3vsTreat1_3	Pyruvate metabolism	24	1	0.481	1	1	0.020
CON_3vsTreat1_3	Glutathione metabolism	26	1	0.509	1	1	0.120
CON_3vsTreat1_3	Aminoacyl-tRNA biosynthesis	67	2	0.544	1	1	0.000
CON_3vsTreat1_3	Valine, leucine and isoleucine degradation	35	1	0.6179	1	1	0.000
CON_3vsTreat1_3	N-Glycan biosynthesis	35	1	0.6179	1	1	0.000
CON_3vsTreat1_3	Pyrimidine metabolism	41	1	0.6771	1	1	0.001
CON_3vsTreat1_3	Purine metabolism	64	1	0.8324	1	1	0.000
CON_3vsTreat2_3	Histidine metabolism	7	1	0.1728	1	1	0.000
CON_3vsTreat2_3	Riboflavin metabolism	7	1	0.1728	1	1	0.000
CON_3vsTreat2_3	Valine, leucine and isoleucine biosynthesis	13	1	0.2977	1	1	0.000
CON_3vsTreat2_3	Sphingolipid metabolism	18	1	0.3876	1	1	0.012
CON_3vsTreat2_3	Citrate cycle (TCA cycle)	20	1	0.4204	1	1	0.027
CON_3vsTreat2_3	Alanine, aspartate and glutamate metabolism	23	1	0.4665	1	1	0.003
CON_3vsTreat2_3	Tryptophan metabolism	23	1	0.4665	1	1	0.375
CON_3vsTreat2_3	Pyruvate metabolism	24	1	0.481	1	1	0.020
CON_3vsTreat2_3	Glycine, serine and threonine metabolism	25	1	0.4952	1	1	0.041
CON_3vsTreat2_3	Aminoacyl-tRNA biosynthesis	67	2	0.544	1	1	0.000
CON_3vsTreat2_3	Tyrosine metabolism	30	1	0.5606	1	1	0.000
CON_3vsTreat2_3	Amino sugar and nucleotide sugar metabolism	34	1	0.607	1	1	0.043
CON_3vsTreat2_3	Arginine and proline metabolism	37	1	0.6387	1	1	0.000
CON_3vsTreat2_3	Purine metabolism	64	1	0.8324	1	1	0.000
CON_3vsTreat4_3	Valine, leucine and isoleucine biosynthesis	13	3	0.0171	1	0.7	1.000
CON_3vsTreat4_3	D-Glutamine and D-glutamate metabolism	5	2	0.0177	1	0.7	1.000
CON_3vsTreat4_3	Riboflavin metabolism	7	2	0.0352	1	0.9258	0.333
CON_3vsTreat4_3	Pantothenate and CoA biosynthesis	12	2	0.096	1	1	0.000
CON_3vsTreat4_3	Glutathione metabolism	26	3	0.1043	1	1	0.135
CON_3vsTreat4_3	Aminoacyl-tRNA biosynthesis	67	5	0.1705	1	1	0.000
CON_3vsTreat4_3	Valine, leucine and isoleucine degradation	35	3	0.2001	1	1	0.000
CON_3vsTreat4_3	Arginine and proline metabolism	37	3	0.2238	1	1	0.249
CON_3vsTreat4_3	Butanoate metabolism	21	2	0.2385	1	1	0.000
CON_3vsTreat4_3	Alanine, aspartate and glutamate metabolism	23	2	0.2722	1	1	0.345
CON_3vsTreat4_3	Nitrogen metabolism	7	1	0.2732	1	1	0.000
CON_3vsTreat4_3	One carbon pool by folate	8	1	0.3057	1	1	0.000
CON_3vsTreat4_3	Vitamin B6 metabolism	8	1	0.3057	1	1	0.333
CON_3vsTreat4_3	Tyrosine metabolism	30	2	0.3889	1	1	0.000
CON_3vsTreat4_3	beta-Alanine metabolism	13	1	0.4481	1	1	0.000
CON_3vsTreat4_3	Folate biosynthesis	16	1	0.5193	1	1	0.000
CON_3vsTreat4_3	Citrate cycle (TCA cycle)	20	1	0.6005	1	1	0.068
CON_3vsTreat4_3	Porphyrin and chlorophyll metabolism	23	1	0.6525	1	1	0.000
CON_3vsTreat4_3	Amino sugar and nucleotide sugar metabolism	34	1	0.7922	1	1	0.043
CON_3vsTreat4_3	Purine metabolism	64	2	0.7928	1	1	0.014

Joint evidence from over-representation and topology (betweenness-centrality) analyses as implemented in MetaboAnalyst (Drosophila melanogaster pathway library). No pathway reached significance after Holm or FDR correction; results are hypothesis-generating.

Table S7a. Larval body weight at seven developmental time points (22–154 h), mean ± SD, n = 10 per group per time point.

Developmental time	Sanjiang (g)	Guanlanhu (g)	Chengmai (g)	Control (g)	SJ vs CON P	GLH vs CON P	CM vs CON P
22 h	0.002 ± 0.001	0.002 ± 0.001	0.002 ± 0.001	0.002 ± 0.001	ns	ns	ns
34 h	0.007 ± 0.002	0.006 ± 0.002	0.006 ± 0.002	0.005 ± 0.002	ns	ns	ns
58 h	0.020 ± 0.004	0.019 ± 0.004	0.018 ± 0.004	0.020 ± 0.005	ns	ns	ns
82 h	0.077 ± 0.008	0.074 ± 0.008	0.072 ± 0.007	0.069 ± 0.009	ns	ns	ns

106 h	0.087 ± 0.007	0.084 ± 0.007	0.081 ± 0.006	0.068 ± 0.008	0.023 *	0.031 *	0.042 *
130 h	0.084 ± 0.006	0.081 ± 0.006	0.078 ± 0.006	0.061 ± 0.007	0.011 *	0.018 *	0.029 *
154 h	0.083 ± 0.007	0.080 ± 0.007	0.077 ± 0.006	0.069 ± 0.008	0.019 *	0.027 *	0.038 *

P values: two-sided t-tests of each laterite group vs the yellow-earth control at the same time point. ns, not significant; * $p < 0.05$, ** $p < 0.01$, *** $p < 0.001$.

Table S7b. Larval body length at the same seven developmental time points, mean ± SD, n = 10 per group per time point.

Developmental time	Sanjiang (mm)	Guanlanhu (mm)	Chengmai (mm)	Control (mm)	SJ vs CON P	GLH vs CON P	CM vs CON P
22 h	2.2 ± 0.4	2.1 ± 0.4	2.0 ± 0.3	2.0 ± 0.3	ns	ns	ns
34 h	5.3 ± 0.7	5.2 ± 0.7	5.1 ± 0.6	5.1 ± 0.6	ns	ns	ns
58 h	10.5 ± 1.1	10.3 ± 1.1	10.1 ± 1.0	10.6 ± 1.2	ns	ns	ns
82 h	15.0 ± 1.3	14.8 ± 1.3	14.6 ± 1.2	15.0 ± 1.4	ns	ns	ns
106 h	15.4 ± 1.2	15.2 ± 1.2	15.0 ± 1.1	16.2 ± 1.3	ns	ns	ns
130 h	15.0 ± 1.0	14.8 ± 1.0	14.6 ± 0.9	17.0 ± 1.5	0.018 *	0.012 *	0.007 **
154 h	14.4 ± 0.9	14.2 ± 0.9	14.0 ± 0.8	16.5 ± 1.4	0.002 **	0.001 **	<0.001 ***

Significance symbols as in Table S7a.

Table S7c. Phenotype endpoints (cryptocephalic-stage onset, pupariation time, wandering depth) and total protein concentration for the three laterite substrates and the yellow-earth control.

Endpoint	Laterite group	Yellow earth (control)	Note
Cryptocephalic stage onset (h)	130	154	≈24 h earlier in laterite
Pupariation time (h)	154	178	24 h earlier in laterite
Post-feeding activity depth (cm)	3–5	8–10	shallower in laterite
Total protein (μg/μL, Sanjiang/Guanlanhu/Chengmai)	7.96 ± 1.44 / 7.67 ± 1.21 / 7.72 ± 1.03 (ns vs control)	7.40 ± 1.45	no significant difference
Growth analysis	linear regression on weight/length vs developmental time (GraphPad Prism)	—	n = 10 larvae randomly sampled per group per time point

Endpoints are given for the merged laterite groups and the yellow-earth control. Total protein: mean ± SD (μg/μL; Sanjiang/Guanlanhu/Chengmai), no significant difference vs control. Growth analysis: linear regression of body weight/length on developmental time (GraphPad Prism); n = 10 larvae randomly sampled per group per time point.

Table S8. Note on the discrepancy between the vendor venn-matrix-based and list-based cross-laterite intersections (Day 1: 2 vs 5 shared DEMs).

Item	Value
Day 1 cross-laterite shared DEMs (vendor venn matrix)	2
Day 1 cross-laterite shared DEMs (name-based intersection of vendor DEM sheets)	5
Suspected cause	Multiple annotations of identical features / compound-naming conventions between vendor files
Handling in this manuscript	Counts from the vendor venn matrix are reported; the name-based intersection is noted here for transparency. Pesticide/drug-like names (e.g., Famprofazone, Trifloxystrobin, Etobenzanid) are treated as probable annotation false positives and excluded from biological interpretation.

Figure S1. Vendor day-resolved patterns of differentially expressed metabolites (DEMs; hypothesis-generating)

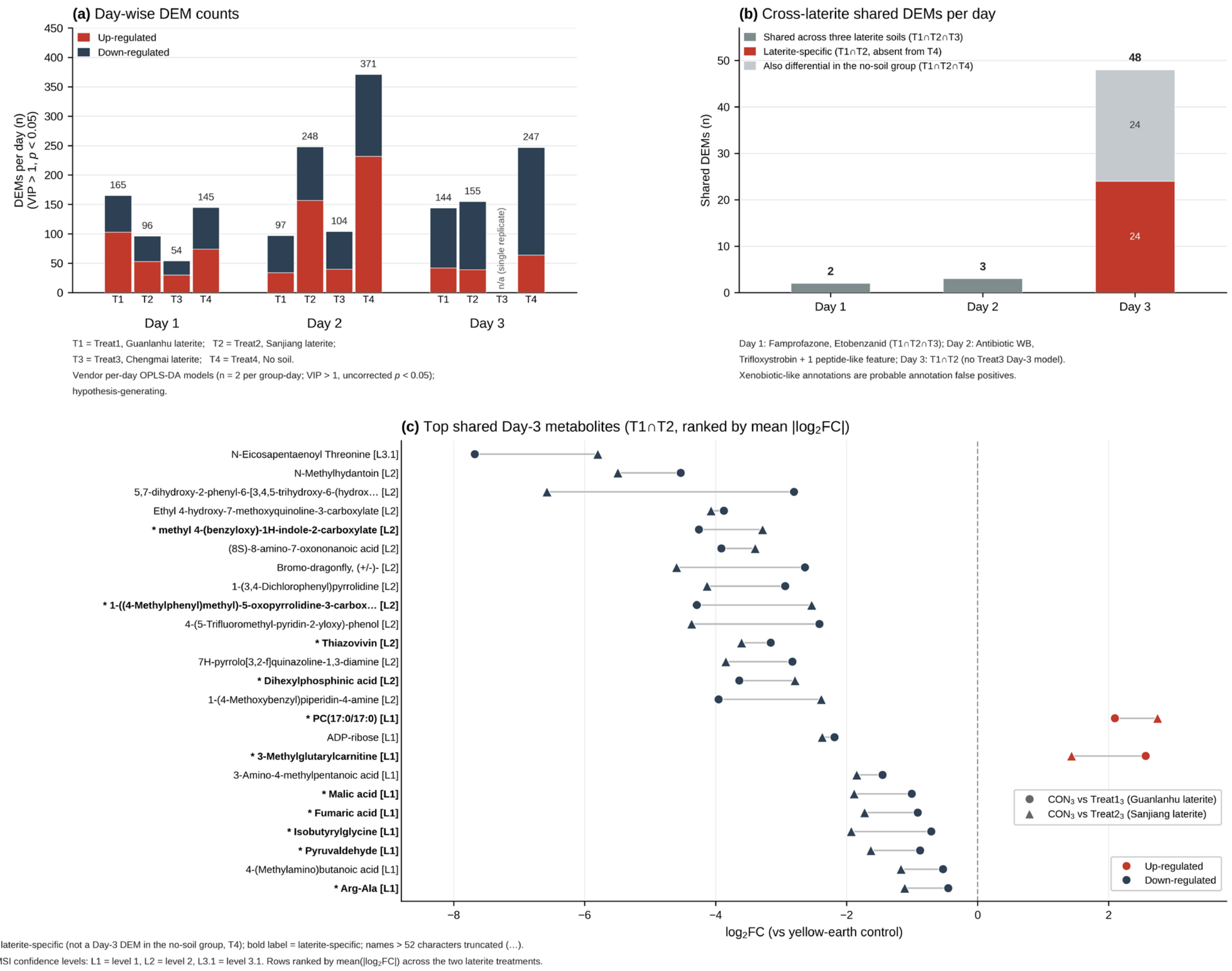

Figure S1. Vendor day-resolved patterns, reported as hypothesis-generating material. (a) Day-wise differentially expressed metabolite (DEM) counts for the four treatment comparisons (VIP > 1 and p < 0.05, unadjusted; n = 2 per group-day; up-regulated in red, down-regulated in blue). No Day-3 model exists for the Chengmai comparison (single replicate). (b) Cross-laterite shared DEMs per day; the Day-3 bar is partitioned into metabolites also differential in the no-soil comparison (Treat1∩Treat2∩Treat4, grey) and laterite-specific metabolites (Treat1∩Treat2\Treat4, red). Day-1 shared DEMs (Famprofazone, Etobenzanid) and Day-2 shared DEMs (Antibiotic WB, Trifloxystrobin, and one peptide-like feature) are xenobiotic-like annotations and probable annotation false positives. (c) Top shared Day-3 metabolites (dumbbell plot; circles = CON_3 vs Treat1_3 Guanlanhu, triangles = CON_3 vs Treat2_3 Sanjiang; red = up, blue = down vs yellow-earth control). MSI level is given in brackets ([L1]/[L2]/[L3.1]); bold labels with an asterisk denote laterite-specific metabolites. Duplicate annotations of identical features were collapsed (the PC(17:0/17:0) triplet and Caplamin/3-amino-4-methylpentanoic acid). All day-wise results rest on n = 2 per group-day without multiple-testing correction and are hypothesis-generating only.
